# The pentapeptide Gly-Thr-Gly-Lys-Thr confers sensitivity to anti-cancer drugs by inhibition of CAGE binding to GSK3β and decreasing the expression of cyclinD1

**DOI:** 10.18632/oncotarget.14621

**Published:** 2017-01-13

**Authors:** Youngmi Kim, Hyuna Kim, Deokbum Park, Hansoo Lee, Yun Sil Lee, Jongseon Choe, Young Myeong Kim, Doyong Jeon, Dooil Jeoung

**Affiliations:** ^1^ Department of Biochemistry, College of Natural Sciences, Kangwon National University, Chunchon 24341, Korea; ^2^ Department of Biological Sciences, College of Natural Sciences, Kangwon National University, Chunchon 24341, Korea; ^3^ College of Pharmacy, Ewha Womans University, Seoul 03760, Korea; ^4^ Graduate School of Medicine, Kangwon National University, Chunchon 24341, Korea; ^5^ L-Base Company, Seoul 01062, Korea

**Keywords:** anti-cancer drug-resistance, CAGE, cyclinD1, GSK3β, peptides

## Abstract

We previously reported the role of cancer/testis antigen CAGE in the response to anti-cancer drugs. CAGE increased the expression of cyclinD1, and pGSK3β^Ser9^, an inactive GSK3β, while decreasing the expression of phospho-cyclinD1Thr^286^. CAGE showed binding to GSK3β and the domain of CAGE (amino acids 231–300) necessary for binding to GSK3β and for the expression regulation of cyclinD1 was determined. ^269^GTGKT^273^ peptide, corresponding to the DEAD box helicase domain of CAGE, decreased the expression of cyclinD1 and pGSK3β^Ser9^ while increasing the expression of phospho-cyclinD1^Thr286^. GTGKT peptide showed the binding to CAGE and prevented CAGE from binding to GSK3β. GTGKT peptide changed the localization of CAGE and inhibited the binding of CAGE to the promoter sequences of cyclin D1. GTGKT peptide enhanced the apoptotic effects of anti-cancer drugs and decreased the migration, invasion, angiogenic, tumorigenic and metastatic potential of anti-cancer drug-resistant cancer cells. We found that Lys^272^ of GTGKT peptide was necessary for conferring anti-cancer activity. Peptides corresponding to the DEAD box helicase domain of CAGE, such as AQTGTGKT, QTGTGKT and TGTGKT, also showed anti-cancer activity by preventing CAGE from binding to GSK3β. GTGKT peptide showed *ex vivo* tumor homing potential. Thus, peptides corresponding to the DEAD box helicase domain of CAGE can be developed as anti-cancer drugs in cancer patients expressing CAGE.

## INTRODUCTION

CAGE, a cancer/testis gene, was isolated by the screening of recombinant cDNA expression libraries using the sera of patients with gastric cancers [1]. A positive rate of anti-CAGE antibody in 7 of 13 (53.8%) patients with microsatellite instability-positive endometrial cancer and in 1 of 3 patients with atypical endometrial hyperplasia [2] suggests that CAGE can be useful for prognosis or early diagnosis of patients with microsatellite instability-positive endometrial cancers. The methylation of CAGE promoter sequences in premalignant lesions suggests that the expression status of CAGE can be a useful diagnostic marker for early detection of cancer [3]. CAGE promotes cell motility by activating extracellular signal-regulated kinase (ERK) and p38 MAPK [4].

CAGE displays oncogenic potential and enhances cellular proliferation by increasing the expression of cyclinD1 via AP-1 [5]. CAGE, through binding to HDAC2, represses the expression of P53 to confer resistance to anti-cancer drugs [6]. miR-200b decreases the expression of cyclinD1 and myosin heavy chain (MHC) [7]. miR-200b confers sensitivity to anti-cancer drugs by decreasing the expression of CAGE [8].

The inhibition of GSK3β ameliorates cisplatin-induced cytotoxicity [9]. The activation of GSK3β increases the phosphorylation of RNPC1 and the expression of p53 [10]. The inactivation of GSK3β leads to the accumulation of mutant p53 [11]. GSK3β suppresses breast carcinogenesis in a manner associated with its effect on the phosphorylation, ubiquitination and the stability of progesterone receptor A [12]. Over-expression of inactive GSK3β inhibits JNK activation, resulting in a suppression of apoptosis by quercetin [13]. GSK3β enhances growth suppression by directly phosphorylating KLF-6 [14]. The mechanisms of radioprotection by GSK3β inhibitors in hippocampal neurons involve the regulation of p53 accumulation [15].

DIF-1 inhibits the growth of HCT-116- and HeLa-xenograft tumors together with decreasing phosphorylation level of GSK3β^Ser9^ and also suppresses the expressions of cyclinD1 [16]. mTORC2 blockade is responsible for reduction of cyclinD1 by GSK3β [17]. Inactive GSK3β attenuates proteasomal degradation of cyclinD1 by reducing phospho-cyclinD1^Thr286^ levels [18]. Inactive GSK3β increases cyclinD1 gene transcription by increasing its transcription factor β-catenin in the nucleus [18].

NF-κB-activating selectable peptides (NASPs), when co-expressed with oncogenic Ras (H-Ras (V12)), allows rodent fibroblasts to overcome senescence and acquire a transformed tumorigenic phenotype [19]. Tumor homing and penetrating peptide iRGD enhances the effect of paclitaxel-loaded PEG-PLA nanoparticles (MT1-NP-PTX) on glioblastoma [20]. Angiotensin-(1-7) [Ang-(1-7)], an endogenous heptapeptide hormone of the renin-angiotensin system displays anti-tumor activity [21]. VEGFR-3 inhibiting peptides display anti-oncogenic activity [22]. The heptapeptide of the PKC delta-V5 region sensitizes human cancer cells through its binding to HSP27 [23]. Inherbin3, a peptide antagonist of ErbB1 receptor, inhibits EGF-induced ErbB1 phosphorylation, cell growth, and migration in two human tumor cell lines [24]. These reports suggest that peptides originating from proteins can be employed as anti-cancer drugs.

We were interested to examine the mechanism of anti-cancer drug-resistance conferred by CAGE.We present evidence that CAGE confers anti-cancer drug-resistance by binding to GSK3β and increasing the expression of cyclin D1 and pGSK3β^Ser9^. We show anti-cancer activity of peptides corresponding to the DEAD box helicase domain of CAGE and the mechanisms of anti-cancer drug sensitivity conferred by these peptides. Thus, peptides corresponding to the DEAD box helicase domain of CAGE can be developed as anti-cancer drugs for the treatment of cancer patients expressing CAGE.

## RESULTS

### CAGE increases the expression of cyclin D1 and pGSK3β^Ser9^ while decreasing the expression of phospho-cyclinD1^Thr286^

CAGE increases the expression of cyclinD1 via AP-1 and E2F [5]. We first investigated the mechanisms involved in the expression regulation of cyclinD1 by CAGE. Anti-cancer drug-resistant cancer cell lines, such as SNU387^R^ and Malme3M^R^ cell lines, showed higher expression level of CAGE than their sensitive counterparts (Supplementary Figure 1A). Meljuso and Hep3B cell lines that do not express CAGE did not show the expression of cyclinD1 (Supplementary Figure 1A). The expression level of CAGE showed an inverse relationship with the expression level of phospho-cyclinD1^Thr286^ and pGSK3β^Tyr216^, an active form of GSK3β (Supplementary Figure 1A). Immunofluorescence staining showed mostly nuclear localization of phospho-cyclinD1^Thr286^ in Malme3M cells, but not in Malme3M^R^ cells (Supplementary Figure 1B). Phosphorylation of GSK3β at Ser9 leads to the increased expression of cyclinD1 [18]. Because the expression level of CAGE showed correlation with that of cyclinD1 (Supplementary Figure 1A), we examined the effect of CAGE on the expression of pGSK3β^Ser9^, an inactive GSK3β. The down-regulation of CAGE decreased the expression of cyclinD1 and pGSK3β^Ser9^ while increasing the expression of phospho-cyclinD1^Thr286^ (Supplementary Figure 1C). Immunofluorescence staining confirmed the effect of the down-regulation of CAGE on the expression of pGSK3β^Ser9^ and phospho-cyclinD1^Thr286^ (Supplementary Figure 1D). CAGE showed the localization in both nucleus and cytoplasm in Malme3M^R^ cells (Supplementary Figure 1D). These results suggest that CAGE increases the expression cyclinD1 in association with its effect on the phosphorylation of GSK3β.

### GSK3β-cyclinD1 axis functions downstream of CAGE

We next examined the relationship between GSK3β and the expression of cyclinD1. The inactivation of GSK3β by LiCl increased the expression of pGSK3β^Ser9^ and cyclinD1 while decreasing the expression of phospho-cyclinD1^Thr286^ in SNU387 and Malme3M cells (Supplementary Figure 2A). However, LiCl did not change the expression level of CAGE (Supplementary Figure 2A). The down-regulation of cyclinD1 did not change the expression of CAGE, phospho-cyclinD1^Thr286^ or pGSK3β^Ser9^ in SNU387^R^ or Malme3M^R^ cells (Supplementary Figure 2B). This suggests that cyclinD1 functions downstream of GSK3β. The down-regulation of cyclinD1 enhanced cleavage of PARP and FAK in response to taxol in Malme3M^R^ cells (Supplementary Figure 2C). The down-regulation of cyclinD1 enhanced sensitivity to taxol in Malme3M^R^ cells (Supplementary Figure 2D). Because CAGE the expression of pGSK3β^Ser9^ (Supplementary Figure 1C), we examined the possibility of the binding of CAGE to GSK3β. The expression of pGSK3β^Tyr216^, active form of GSK3β, was lower in Malme3M^R^ cells than in Malme3M cells (Supplementary Figure 2E). CAGE showed binding to GSK3β in SNU387^R^ (data now shown) and Malme3M^R^ cells (Supplementary Figure 2E). CAGE showed a co-localization with GSK3β in SNU387^R^ (Supplementary Figure 2F) and Malme3M^R^ cells (data not shown). It remains to be seen whether CAGE exerts a direct regulation on GSK3β activity. These results suggest that CAGE confers resistance to anti-cancer drugs through GSK3β-cyclinD1 axis.

### CAGE domain encompassing amino acids 231-300 is necessary for binding of CAGE to GSK3β and for increasing the expression of cyclinD1

**Figure 1:**
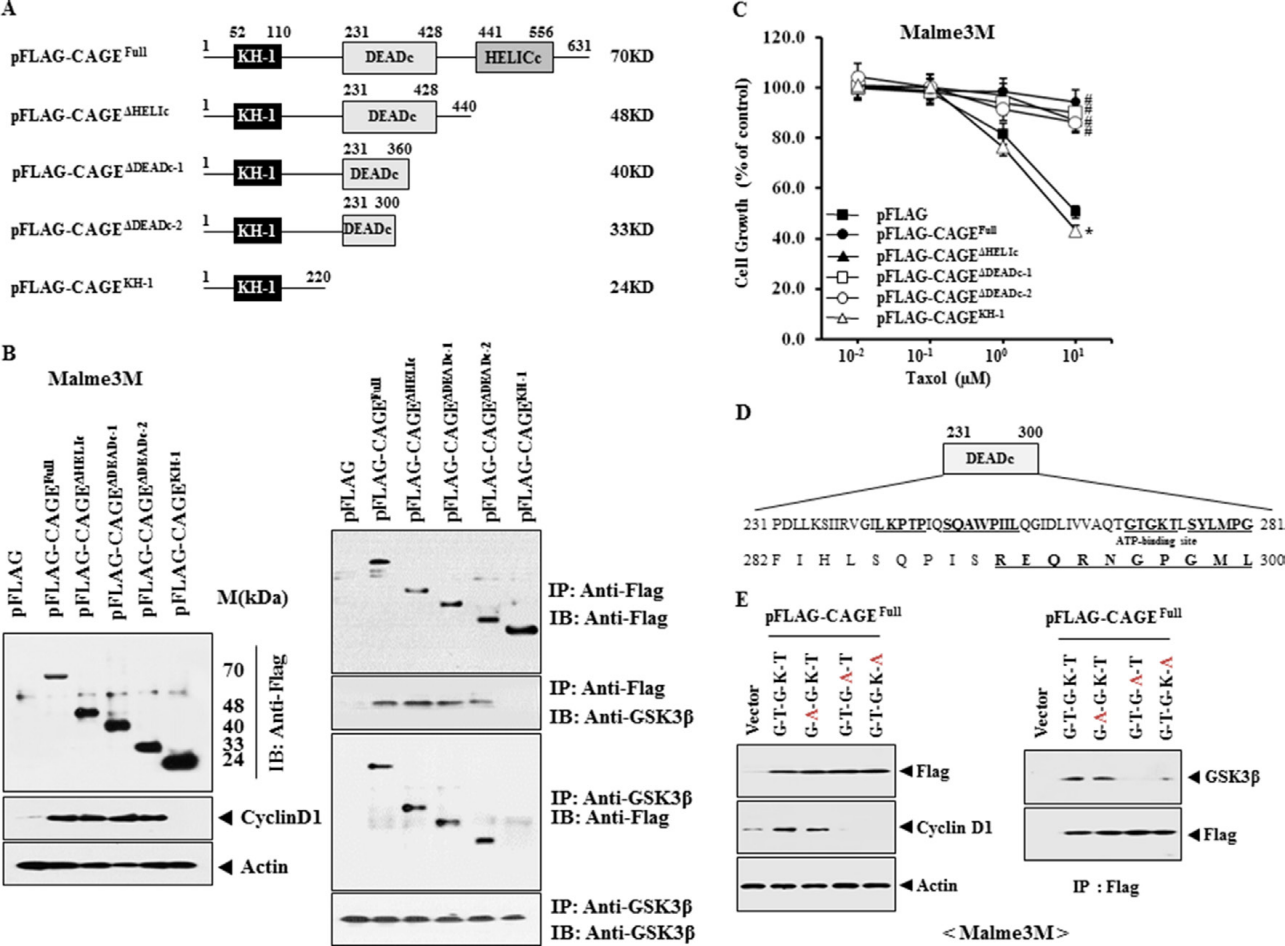
CAGE domain encompassing amino acids 231-300 is necessary for binding of CAGE to GSK3β and for increasing the expression of cyclinD1 (**A**) Shows a series of CAGE deletion constructs. FL denotes full-length. (**B**) Malme3M cells were transiently transfected with each CAGE construct (1 μg). At 48 h after transfection, cell lysates were subjected to Western blot analysis (left panel). Cell lysates were immunoprecipitated with the indicated antibody (2 μg/ml), followed by Western blot analysis (right panel). A representative of at least two reproducible results is shown. (**C**) Malme3M cells were transiently transfected with each CAGE construct (1 μg). At 24 h after transfection, cells were treated with various concentrations of taxol for 24 h, followed by MTT assays. The mean ± S.E. of three independent experiments is shown. *p < 0.05; #*p* < 0.05. *; comparison was made between Malme3M cells transfected with CAGE^KH-1^ deletion construct and the same cells transfected with CAGE^Full^. #; comparison was made between Malme3M cells transfected with the indicated construct and the same cells transfected with pFLAG. (**D**) Shows putative binding sites within the DEAD box domain of CAGE. The bold underlined peptides denote peptides with potential tumor homing activity. (**E**) Malme3M cells were transiently transfected with the indicated construct (1 μg). At 48 h after transfection, cell lysates were subjected to immunoprecipitation and Western blot analysis. A representative of at least two reproducible results is shown.

Because CAGE showed binding to GSK3β (Supplementary Figure 2E), we wanted to determine the domain of CAGE that is necessary for binding to GSK3β. We hypothesized that this domain would be critical for conferring resistance to anti-cancer drugs. For this, various CAGE deletion constructs were made (Figure 1A). We found that the CAGE domain corresponding to amino acids 231-300 was necessary for increasing the expression of cyclinD1 and binding to GSK3β (Figure 1B). CAGE domain corresponding to amino acids 231-300 was also necessary for conferring resistance to taxol (Figure 1C). CAGE domain (amino acids 231-300) contains ATP-binding site (^269^GTGKT^273^), and database (TumorHOPe) search predicts that GTGKT, SQAWP and RNGPG peptides display tumor homing potential (Figure 1D). TumorHOPe database shows that GTG-containing peptides, such as, CWGTGLC and WGTGLC peptides, display homing potential to mouse melanoma based on phage display. We hypothesized that ATP-binding site of CAGE would be necessary for binding to GSK3β and for increasing the expression of cyclinD1. We introduced point mutation into ATP-binding site of CAGE to examine the importance of ATP-binding site for increasing the expression of cyclinD1 and the binding of CAGE to GSK3β. Full-length wild type CAGE (G-T-G-K-T) and mutant CAGE (G-A-G-K-T) increased the expression of cyclinD1 and showed binding to GSK3β in Malme3M cells (Figure 1E). However, full-length point mutant CAGEs, such as G-T-G-A-T and G-T-G-K-A, did not increase the expression of cyclinD1 or binding to GSK3β in Malme3M cells (Figure 1E). This suggests the importance of ATP-binding site of CAGE (K272, T273) in the expression of cyclinD1 and the binding of CAGE to GSK3β. We wanted to examine the mechanism of the increased expression of cyclinD1 by CAGE. CyclinD1 promoter contains the binding sites for various transcriptional factors such as HDAC2, SNAIL, AP1 and SP1 (Supplementary Figure 3A). Because cyclinD1 promoter sequences contain binding site for HDAC2, we hypothesized that CAGE would bind to the promoter sequences of cyclinD1 to increase the expression of cyclinD1. Malme3M cells transfected with full-length wild type CAGE or CAGE deletion construct (pFLAG-CAGE^ΔHELIc^, pFLAG-CAGE^ΔDEADc-1^ or FLAG-CAGE^ΔDEADc-2^) showed the binding of CAGE to the promoter sequences of cyclinD1 (Supplementary Figure 3B). However, Malme3M cells transfected with CAGE^KH-1^ deletion construct did not show the binding of CAGE to the promoter sequences of cyclinD1 (Supplementary Figure 3B). Malme3M cells transfected with full-length wild type CAGE (G-T-G-K-T) or full-length point mutant CAGE (G-A-G-K-T) showed the binding of CAGE to the promoter sequences of cyclinD1 (Supplementary Figure 3C). However, Malme3M cells transfected with full-length point mutant CAGEs, such as G-T-G-A-T and G-T-G-K-A, did not show the binding of CAGE to the promoter sequences of cyclinD1 (Supplementary Figure 3C). These results suggest that the increased expression of cyclinD1 by CAGE involves the direct binding of CAGE to the promoter sequences of cyclinD1. Full-length mutant CAGEs, such as G-T-G-A-T and G-T-G-K-A, did not confer resistance to anti-cancer drugs in Malme3M cells (Supplementary Figure 4A). Unlike wild type full-length CAGE and mutant CAGE mutant (G-A-G-K-T), full-length mutant CAGEs, such as G-T-G-A-T and G-T-G-K-A, increased caspase-3 activity in response to taxol in Malme3M cells (Supplementary Figure 4B). G-T-G-A-T and G-T-G-K-A mutants induced cleavage of PARP in response to taxol in Malme3M cells (Supplementary Figure 4C). Unlike wild type full-length CAGE and mutant CAGE mutant (G-A-G-K-T), full-length mutants G-T-G-A-T and G-T-G-K-A did not enhance the migration or invasion potential of Malme3M cells (Supplementary Figure 4D). Malme3M cells transfected with wild type full-length CAGE or mutant CAGE mutant (G-A-G-K-T) showed a higher growth potential than Malme3M cells transfected with full-length mutant G-T-G-A-T or G-T-G-K-A based on colony formation assay (Supplementary Figure 4E). These results suggest that CAGE domain encompassing amino acids 231-300 is necessary for binding to GSK3β and for the increased expression of cyclinD1. These results provide the basis for designing anti-cancer peptides corresponding to the DEAD box helicase domain of CAGE.

### GTGKT peptide decreases the expression of cyclinD1, pGSK3β^Ser9^ and inhibits the binding of CAGE to GSK3β

**Figure 2:**
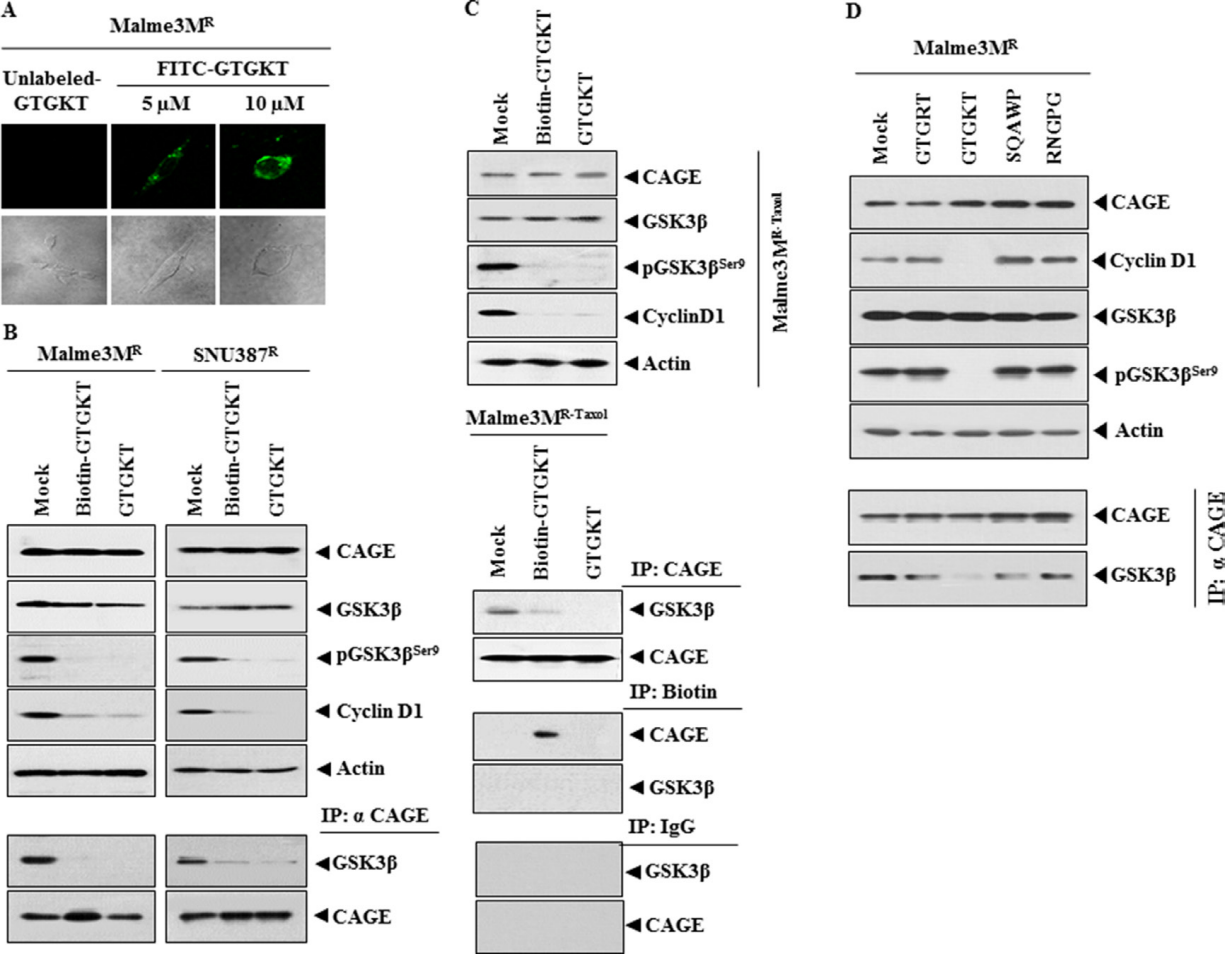
GTGKT peptide decreases the expression of cyclinD1, pGSK3β^Ser9^ and inhibits the binding of CAGE to GSK3β (**A**) Malme3M^R^ cells were treated with FITC-labeled GTGKT peptide (5, 10 μM) or unlabeled GTGKT peptide (10 μM). At 48 h after treatment, the expression of GTGKT peptide was determined. (**B**) Malme3M^R^ or SNU387^R^ cells were treated with the indicated peptide (10 μM). At 48 h after treatment, cell lysates were subjected to Western blot analysis Cell lysates prepared were subjected to immunoprecipitation, followed by Western blot analysis (lower panel). A representative of at least two reproducible results is shown. (**C**) Malme3M^R-taxol^ cells were treated with the indicated peptide (10 μM). At 48 h after treatment, cell lysates prepared were subjected to Western blot analysis. Cell lysates prepared were subjected to immunoprecipitation, followed by Western blot analysis (lower panel). A representative of at least two reproducible results is shown. (**D**) The indicated cancer cells were treated with the indicated peptide (10 μM). At 48 h after treatment, cell lysates prepared were subjected to immunoprecipitation and Western blot analysis. A representative of at least two reproducible results is shown.

^269^GTGKT^273^ amino acids within the DEAD box domain correspond to the ATP-binding site of CAGE and are predicted to display tumor homing potential. We therefore examined the potential of GTGKT peptide as anti-cancer peptide in association with its potential effect on the expression of cyclinD1 and the binding of CAGE to GSK3β. FITC-labeled GTGKT peptide showed expression in Malme3M^R^ cells (Figure 2A). GTGKT and biotin-GTGKT peptide decreased the expression of cyclinD1 and pGSK3β^Ser9^, but not the expression of CAGE (Figure 2B). GTGKT and biotin-GTGKT peptide inhibited the binding of CAGE to GSK3β in SNU387^R^ and Malme3M^R^ cells (Figure 2B). GTGKT or biotin-labeled GTGKT peptide did not change the expression of CAGE in Malme3M^R-Taxol^ cell line, an anti-cancer drug-resistant cancer cell line made resistant to taxol (Figure 2C). GTGKT and biotin-labeled GTGKT peptide decreased the expression of cyclinD1 and pGSK3β^Ser9^, and inhibited the binding of CAGE to GSK3β in Malme3M^R-Taxol^ cell line (Figure 2C). Biotin-labeled GTGKT peptide showed binding to CAGE, but not to GSK3β in Malme3M^R-Taxol^ cells (Figure 2C) and Malme3M^R^ cells (data shown). GTGKT, but not SQAWP or RNGPG peptide, decreased the expression of cyclinD1 in Malme3M^R^ cells (Figure 2D). GTGKT, but not SQAWP or RNGPG peptide within DEAD box domain of CAGE (amino acids 231-300), inhibited the binding of CAGE to GSK3β in Malme3M^R^ cells (Figure 2D). We therefore focused on GTGKT peptide throughout this study. These results suggest that GTGKT peptide displays anti-cancer activity by inhibiting the binding of CAGE to GSK3β in anti-cancer drug-resistant cancer cells.

### GTGKT peptide binding to CAGE is necessary for conferring sensitivity to anti-cancer drugs

Because GTGKT peptide inhibited the binding of CAGE to GSK3β (Figure 2B and 2C), we examined the importance of GTGKT peptide binding to CAGE or GSK3β. For this, full-length CAGE or each CAGE deletion construct was transfected into Malme3M Cells. Cells were then treated with GTGKT peptide. GTGKT peptide showed binding to full-length CAGE (Supplementary Figure 5A). However, GTGKT peptide did not show binding to any other CAGE deletion constructs (Supplementary Figure 5A). This suggests that the binding domain of GTGKT resides in helicase domain of CAGE (amino acids 441-631). GTGKT peptide did not show binding to GSK3β (Supplementary Figure 5A). We next examined whether GTGKT binding to CAGE would be necessary for enhancing the response to anti-cancer drugs. GTGKT peptide conferred sensitivity to taxol in Malme3M cells transfected with full-length CAGE (Supplementary Figure 5B). CAGE deletion constructs, such as pFLAG-CAGE^ΔHELIc^, pFLAG-CAGE^ΔDEADc-1^ and pFLAG-CAGE^ΔDEADc-2^, conferred resistance to taxol in Malme3M cells (Supplementary Figure 5B). GTGKT peptide did not enhance caspase-3 activity in Malme3M cells transfected with pFLAG-CAGE^ΔHELIc^, pFLAG-CAGE^ΔDEADc-1^ or pFLAG-CAGE^ΔDEADc-2^ construct in response to celastrol or taxol (Supplementary Figure 5C). GTGKT peptide did not induce cleavage of PARP in Malme3M cells transfected with pFLAG-CAGE^ΔHELIc^, pFLAG-CAGE^ΔDEADc-1^ or pFLAG-CAGE^ΔDEADc-2^ construct in response to taxol (Supplementary Figure 5D). Because GTGKT peptide showed binding to CAGE (Supplementary Figure 5A), we examined whether GTGKT peptide showed a co-localization with CAGE. CAGE and FITC-GTGKT peptide showed localization in the nucleus and cytoplasm in Malme3M^R^ cells (Supplementary Figure 6). CAGE showed mostly cytoplasmic localization in Malme3M^R^ cells treated with FITC-GTGKT peptide (Supplementary Figure 6). CAGE showed co-localization with GTGKT peptide in the cytoplasm in Malme3M^R^ cells (Supplementary Figure 6). This implies that GTGKT peptide prevents nuclear localization of CAGE. These results suggest that GTGKT peptide binding to CAGE is necessary for conferring sensitivity to anti-cancer drugs.

### GTGKT peptide changes localization of CAGE to inhibit the binding of CAGE to the promoter sequences of cyclinD1

**Figure 3:**
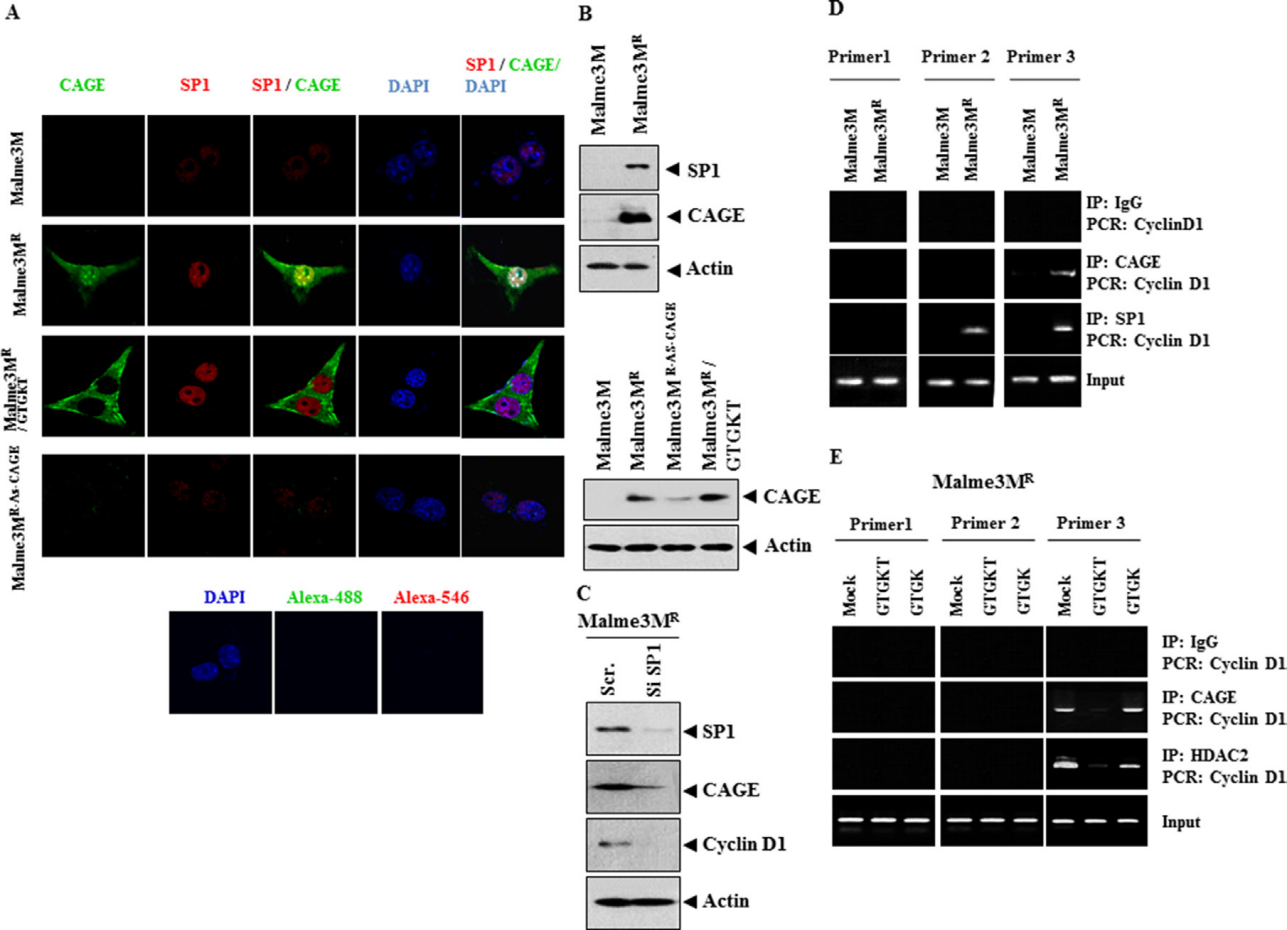
GTGKT peptide changes localization of CAGE to inhibit the binding of CAGE to the promoter sequences of cyclinD1 (**A**) Immunofluorescence staining employing the indicated antibody was performed. Malme3M^R-AS-CAGE^ cells denote Malme3M^R^ cells that stably express anti-sense CAGE. Malme3M^R^ cells were treated with the indicated peptide (10 μM). At 48 h after treatment, cells were then subjected to immunofluorescence staining. (**B**) Cell lysates from the indicated cancer cells were subjected to Western blot analysis (upper panel). Malme3MR cells were treated with the indicated peptide (10 μM). At 48 h after treatment, cell lysates were subjected to Western blot analysis (lower panel). Cell lysates isolated from Malme3M, Malme3M^R^ or Malme3M^R-AS-CAGE^ cells were subjected to Western blot analysis (lower panel). (**C**) Malme3M^R^ cells were transiently transfected with the indicated siRNA (each at 10 nM). At 48 h after transfection, cell lysates were subjected to Western blot analysis. (**D**) Cell lysates isolated from the indicated cancer cells were subjected to ChIP assays. (**E**) Malme3MR cells were treated with the indicated peptide (10 μM). At 48 h after treatment, cell lysates prepared were subjected to ChIP assays.

Because GTGKT peptide inhibited the binding of CAGE to GSK3β (Figure 2A) and also showed binding to CAGE (Figure 2B), we examined whether GTGKT peptide would inhibit the function of CAGE. CAGE showed the binding to the promoter sequences of cyclinD1 (Supplementary Figure 3B). We hypothesized that GTGKT peptide would inhibit the binding of CAGE to the promoter sequences of cyclinD1. We hypothesized that SP1, through binding to CAGE, might increase the expression of cyclinD1. In Malme3M^R^ cells, SP1 showed nuclear localization while CAGE was present in both nucleus and cytoplasm (Figure 3A). CAGE showed a co-localization with SP1 in Malme3M^R^ cells (Figure 3A). Immunofluorescence staining revealed that GTGKT peptide did not change the expression of CAGE or SP1 in (Figure 3A). However, GTGKT peptide changed the localization of CAGE into cytoplasmic, which inhibited co-localization of CAGE with SP1 (Figure 3A). Immunofluorescence staining revealed that Malme3M^R^ cells that stably express anti-sense CAGE (Malme3M^R-As-CAGE^) did not express SP1 (Figure 3A). The expression level of SP1 was higher in Malme3M^R^ cells (Figure 3B). GTGKT peptide did not change the expression of CAGE in Malme3M^R^ cells (Figure 3B). The fact that GTGKT peptide changes the localization of CAGE and decreases the expression of cyclinD1 without changing the expression level of SP1 implies that GTGKT peptide exerts a transcriptional regulation on the expression of cyclinD1. The down-regulation of SP1 decreased the expression of CAGE and cyclin D1 (Figure 3C). The down-regulation of SP1 enhanced the sensitivity to anti-cancer drugs (Supplementary Figure 7A), the cleavage of FAK (Supplementary Figure 7B) and the caspase-3 activity (Supplementary Figure 7C) in response to anti-cancer drugs in Malme3M^R^ cells. The down-regulation of SP1 decreased the invasion and migration potential of Malme3M^R^ cells (Supplementary Figure 7D). Just like CAGE, SP1 showed the binding to the promoter sequences of cyclinD1 in Malme3M^R^ cells (Figure 3D), suggesting the direct expression regulation of cyclinD1 by SP1. GTGKT peptide, but not GTGK peptide, prevented CAGE and HDAC2 from binding to the promoter sequences of cyclinD1 in Malme3M^R^ cells (Figure 3E). CAGE showed the binding to SP1 (data not shown). GTGKT peptide, but not GTGK peptide, inhibited the binding of CAGE to SP1 (data not shown). These results suggest that GTGKT peptide may cause structural changes to CAGE, which in turn inhibits the binding of CAGE to the promoter sequences of cyclinD1.

### GTGKT peptide decreases the tumorigenic potential of anti-cancer drug-resistant cancer cells

**Figure 4:**
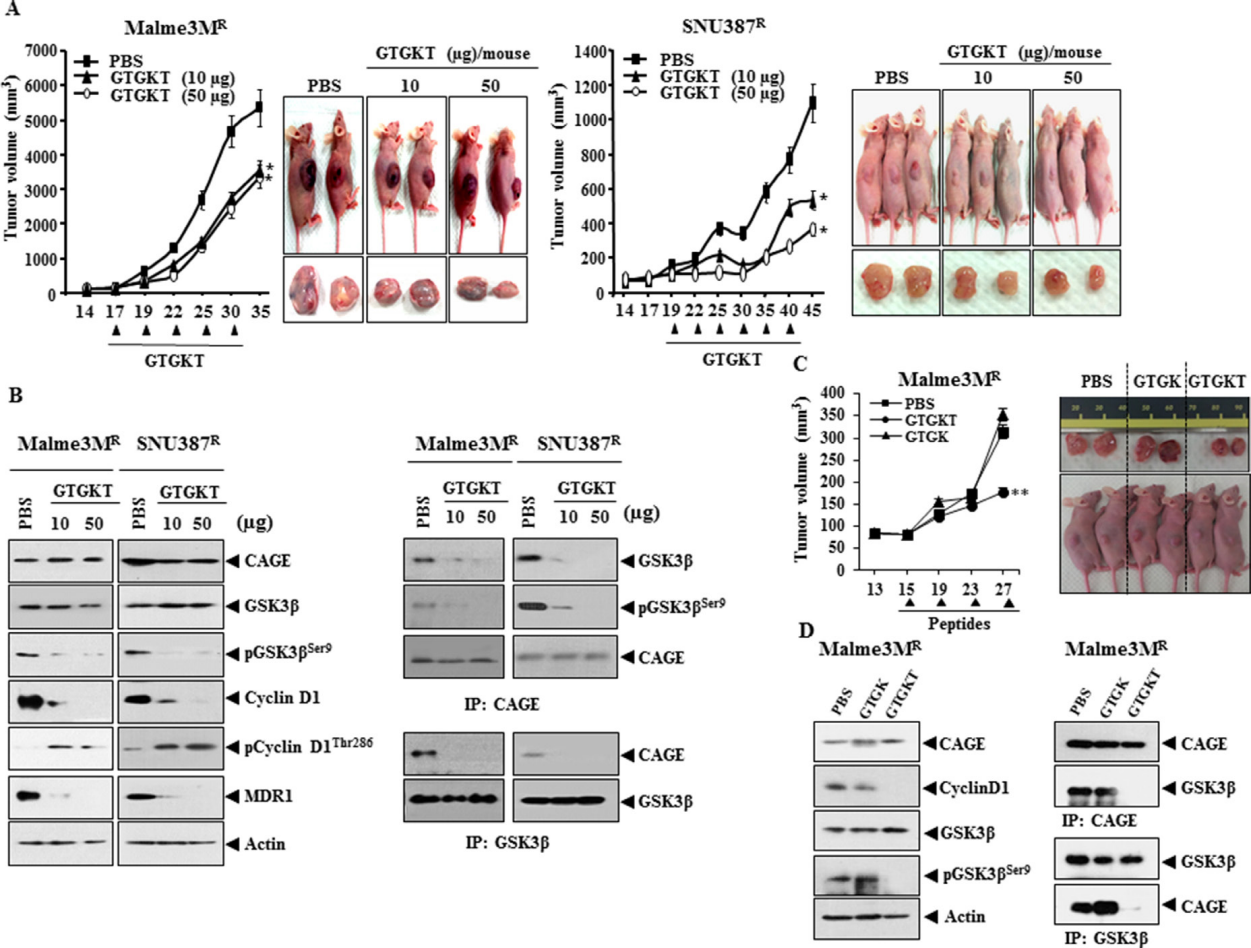
GTGKT peptide decreases the tumorigenic potential of anti-cancer drug-resistant cancer cells (**A**) Malme3M^R^ (1 × 10^6^) or SNU387R (1 × 10^6^) cells were injected into the dorsal flanks of athymic nude mice. Following the establishment of sizeable tumor, GTGKT peptide (10 μg/mouse or 50 μg/mouse) was injected into the tail vein. Each experimental group consisted of five mice. Each value represents an average obtained from the five athymic nude mice of each group. Data are expressed as a mean ± S.D. Each figure shows a representative image of the mice in each group at the time of sacrifice. Comparison was made between group of mice injected with GTGKT peptide (10 μg/mouse or 50 μg/mouse) and group of mice injected with PBS. *p < 0.05. (**B**) Tumor lysates were subjected to Western blot analysis (left panel). Tumor lysates were also immunoprecipitated with the indicated antibody (2 μg/ml), followed by Western blot analysis (right panel). (**C**) Malme3M^R^ (1 × 10^6^) cells were injected into the dorsal flanks of athymic nude mice. Following the establishment of sizeable tumor, GTGKT or GTGK peptide (each at 50 μg/mouse) was injected into the tail vein. Each experimental group consisted of five mice. Comparison was made between group of mice injected with GTGKT peptide (50 μg/mouse) and group of mice injected with GTGK peptide. **p < 0.005. (**D**) Tumor lysates were subjected to Western blot analysis (left panel). Tumor lysates were also immunoprecipitated with the indicated antibody (2 μg/ml), followed by Western blot analysis (right panel).

We first examined the effect of CAGE on the tumorigenic potential and the *in vivo* response to taxol. Malme3M^R-As-CAGE^ cells that stably express anti-sense CAGE showed lower tumorigenic potential than Malme3M^R^ cells and showed higher sensitivity to taxol than Malme3M^R^ cells (Supplementary Figure 8A). The xenograft of Malme3M^R-As-CAGE^ cells showed lower expression level of CAGE, cyclinD1 and pGSK3β^Ser9^ while showing higher expression level of phospho-cyclinD1^Thr286^ than the xenograft of Malme3M^R-vector^ cells (Supplementary Figure 8B). The *in vivo* down-regulation of CAGE by CAGE siRNA also decreased the tumorigenic potential of Malme3M^R^ cells (data not shown). We next examined the effect of GTGKT peptide on the tumorigenic potential of anti-cancer drug-resistant cancer cells. GTGKT peptide decreased the tumorigenic potential of SNU387^R^ and Malme3M^R^ cells (Figure 4A). Western blot of tumor tissue lysates show that GTGKT peptide decreased the expression of cyclinD1, pGSK3β^Ser9^ and MDR1 while increasing the expression of phospho-cyclinD1^Thr286^ (Figure 4B). Immunoprecipitation of tumor lysates showed that GTGKT peptide inhibited the binding of CAGE to GSK3β (Figure 4B). Unlike GTGKT peptide, GTGK peptide did not decrease the tumorigenic potential of Malme3M^R^ cells (Figure 4C), the expression of cyclinD1, pGSK3β^Ser9^ or the binding of CAGE to GSK3β (Figure 4D). These results suggest that GTGKT peptide decreases the tumorigenic potential of cancer cells by decreasing the expression of cyclinD1, pGSK3β^Ser9^ and inhibiting the binding of CAGE to GSK3β.

### GTGKT peptide decreases the metastatic potential of anti-cancer drug-resistant cancer cells

**Figure 5:**
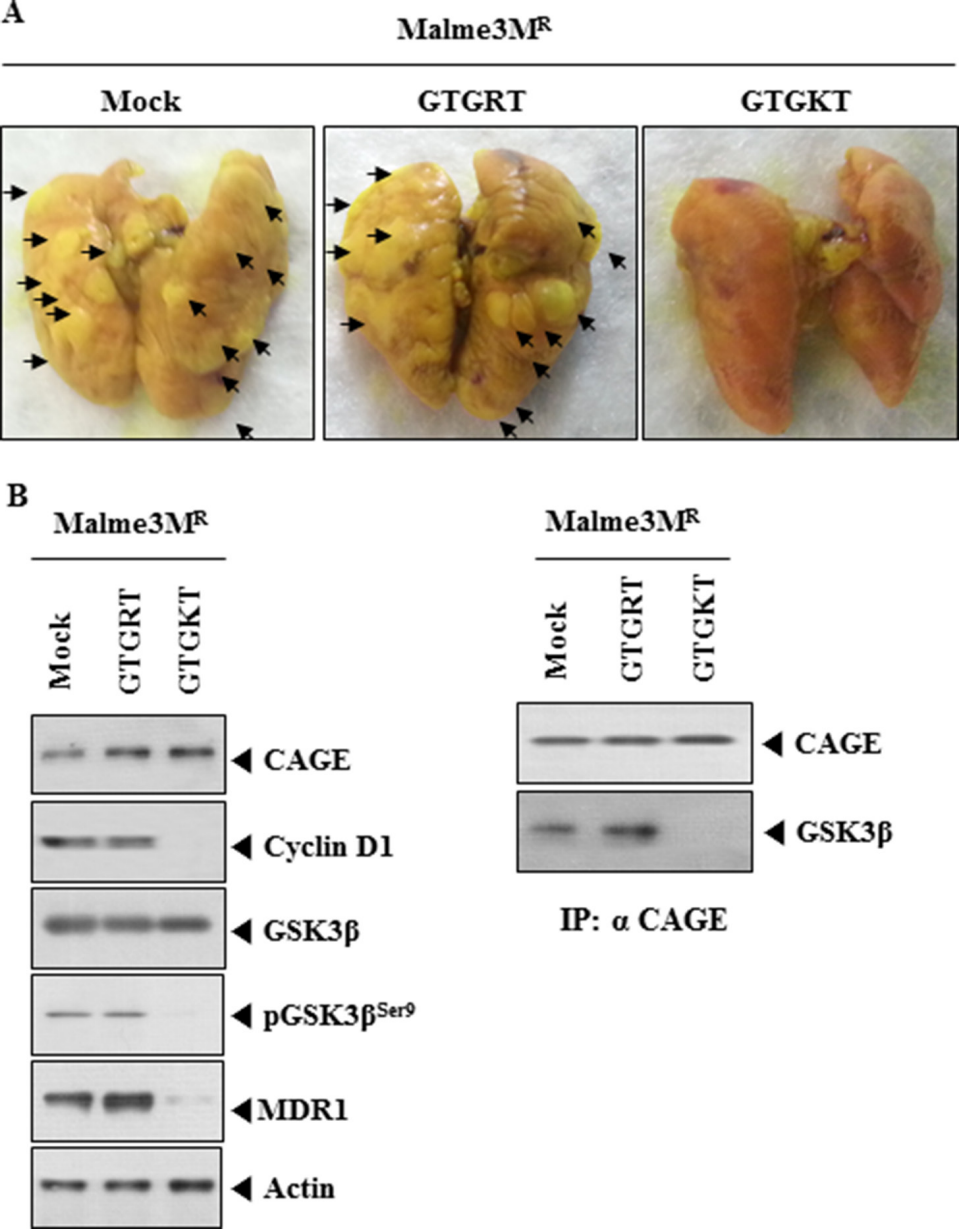
GTGKT peptide decreases the metastatic potential of Malme3M^R^ cells (**A**) Each experimental group consists of five athymic nude mice. Each figure shows a representative image of the mice in each experimental group. Malme3M^R^ Cells (1 × 10^6^) were injected intravenously into the tail vein of 4-week-old athymic nude mice, and the extent of lung metastasis was evaluated. GTGRT peptide or GTGKT peptide (each at 50 μg/mouse) was intravenously injected five times over a total of 4 weeks. (**B**) Lysates were subjected to Western blot analysis (left panel). Tumor lysates were also immunoprecipitated with the indicated antibody (2 μg/ml), followed by Western blot analysis (right panel).

GTGKT peptide, but not GTGRT peptide, decreased the metastatic potential of Malme3M^R^ cells (Figure 5A). GTGKT peptide decreased the metastatic potential of Malme3M^R^ cells in a manner associated with its effect on the binding of CAGE to GSK3β (Figure 5B). Western blot analysis of tumor lysates showed that GTGKT peptide, but not GTGRT peptide, decreased the expression of cyclinD1, pGSK3β^Ser9^ and MDR1 (Figure 5B). These results suggest that GTGKT peptide decreases the metastatic potential of Malme3M^R^ cells by decreasing the expression of cyclinD1 and inhibiting the binding of CAGE to GSK3β.

### Lys^272^ residue of ^269^GTGKT^273^ peptide is necessary for conferring anti-cancer activity

**Figure 6:**
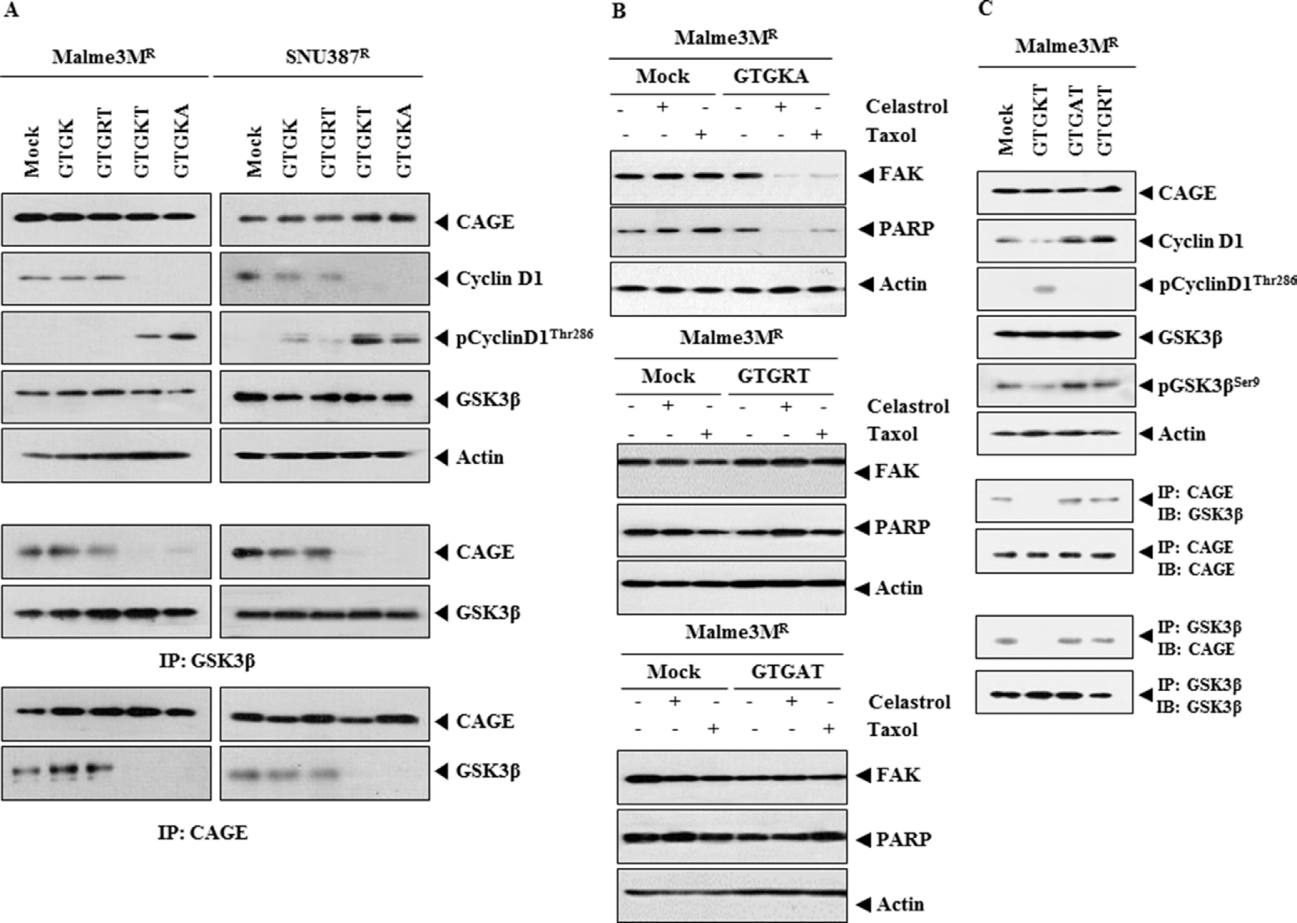
Lys^272^ residue of ^269^GTGKT^273^ peptide is necessary for anti-cancer activity (**A**) Malme3M^R^ or SNU387^R^ cells were treated with the indicated peptide (each at 10 μM). At 48 h after transfection, cell lysates were subjected to Western blot analysis. Cell lysates were also immunoprecipitated with the indicated antibody (2 μg/ml), followed by Western blot analysis. (**B**) Malme3M^R^ cells were treated with the indicated peptide (each at 10 μM). At 24 h after treatment, cells were treated with celastrol (1 μM) or taxol (1 μM) for 24 h. Cell lysates were then subjected to Western blot analysis. (**C**) Malme3M^R^ cells were treated with the indicated peptide (each at 10 μM). At 48 h after treatment, cell lysates were subjected to Western blot analysis. Cell lysates were also immunoprecipitated with the indicated antibody (2 μg/ml), followed by Western blot analysis.

We wanted to determine residues of GTGKT peptide necessary for conferring sensitivity to anti-cancer drugs. GTGK peptide did not change the expression of cyclinD1, phospho-cyclinD1^Thr286^ or the binding of CAGE to GSK3β (Figure 6A). This suggests that the length of peptide corresponding to the DEAD box helicase domain of CAGE is necessary for conferring anti-cancer activity. GTGRT peptide did not change the expression level of cyclinD1, phospho-cyclinD1^Thr286^ or the binding of CAGE to GSK3β (Figure 6A). GTGKT and GTGKA peptides decreased the expression of cyclinD1 while increasing the expression of phospho-cyclinD1^Thr286^ and inhibiting the binding of CAGE to GSK3β (Figure 6A). GTGKA peptide, but not GTGRT or GTGAT peptide, induced cleavage of PARP and FAK in response to celastrol and taxol in Malme3M^R^ cells (Figure 6B). GTGKT peptide, but not GTGRT or GTGAT peptide, decreased the expression of cyclinD1, and pGSK3β^Ser9^ while increasing the expression of phospho-cyclinD1^Thr286^ and inhibited the binding of CAGE to GSK3β (Figure 6C). GAGKT peptide did not change the expression level of cyclinD1 in Malme3M^R^ cells (data not shown). These results suggest that Lys^272^ residue of GTGKT peptide is necessary for conferring anti-cancer activity.

### TGTGKT, QTGTGKT and AQTGTGKT peptides enhance apoptotic effects of anti-cancer drugs

**Figure 7:**
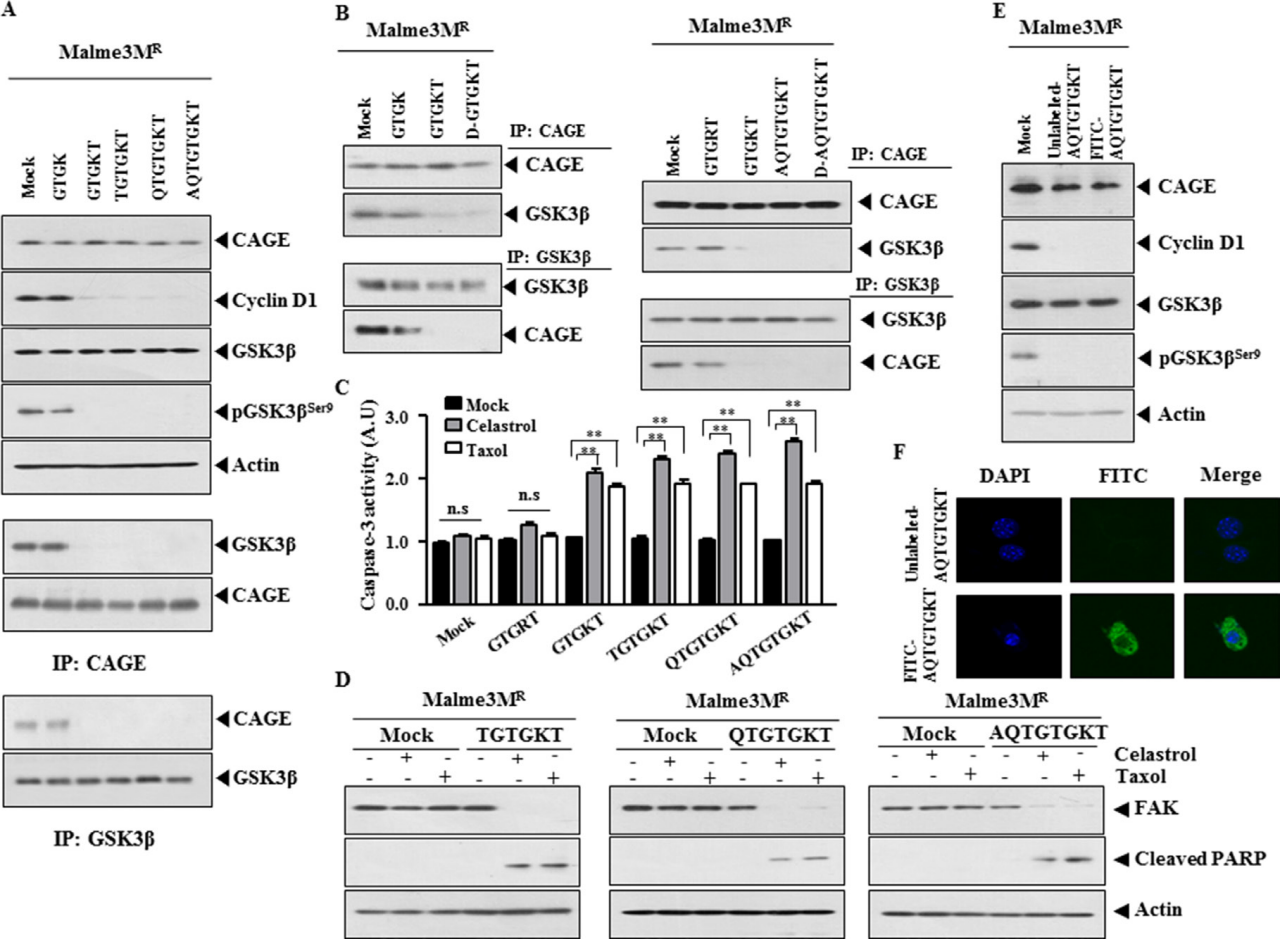
TGTGKT, QTGTGKT and AQTGTGKT peptides enhance apoptotic effects of anti-cancer drugs (**A**) Malme3M^R^ cells were treated with the indicated peptide (each at 10 μM). At 48 h after treatment, cell lysates were subjected to Western blot analysis. Cell lysates were also immunoprecipitated with the indicated antibody (2 μg/ml), followed by Western blot analysis. A representative of at least two reproducible results is shown. (**B**) Malme3M^R^ cells were treated with the indicated peptide (each at 10 μM). At 48 h after treatment, immunoprecipitation employing the indicated antibody (2 μg/ml) was performed. (**C**) Malme3M^R^ cells were treated with the indicated peptide (each at 10 μM). At 24 h after treatment, cells were then treated with celastrol (1 μM) or taxol (1 μM) for 24 h, followed by caspase-3 activity assays. **p < 0.005. (**D**) Malme3M^R^ cells were treated with TGTGKT, QTGTGKT or AQTGTGKT peptide (each at 10 μM). At 24 h after treatment, cells were then treated with celastrol (1 μM) or taxol (1 μM) for 24 h, followed by caspase-3 activity assays. (**E**) Malme3M^R^ cells were treated with the indicated peptide (each at 10 μM). At 48 h after treatment, cell lysates were subjected to Western blot analysis. (**F**) FITC-labeled AQTGTGKT peptide (10 μM) or AQTGTGKT peptide (10 μM) was added to Malme3M^R^ cells. At 48 h after transfection, the expression of FITC-labeled AQTGTGKT peptide was determined.

We examined the anti-cancer activity of other peptides corresponding to the DEAD box helicase domain of CAGE. ^268^TGTGKT^273^, ^267^QTGTGKT^273^ and ^266^AQTGTGKT^273^ peptides decreased the expression of cyclin D1 and pGSK3β^Ser9^ and inhibited the binding of CAGE to GSK3β (Figure 7A). ^266^AQTGTGKT^273^ peptide prevented the binding of CAGE to the promoter sequences of cyclinD1 based on ChIP assays (data not shown). D-GTGKT and D-AQTGTGKT peptides inhibited the binding of CAGE to GSK3β (Figure 7B). GTGKT, TGTGKT, QTGTGKT and AQTGTGKT peptides, but not GTGRT peptide, increased caspase-3 activity in response to celastrol and taxol in Malme3M^R^ cells (Figure 7C). TGTGKT, QTGTGKT and AQTGTGKT peptides induced the cleavage of PARP and FAK in response to celastrol and taxol (Figure 7D). AQTGTGKT and FITC-labeled AQTGTGKT peptides decreased the expression of cyclinD1 and pGSK3β^Ser9^ in Malme3M^R^ cells (Figure 7E). FITC-labeled AQTGTGKT peptide showed expression in Malme3M^R^ cells (Figure 7F). These results indicate that peptides within DEAD box domain of CAGE enhance apoptotic effects of anti-cancer drugs by decreasing the expression of cyclinD1, pGSK3β^Ser9^ and the binding of CAGE to GSK3β.

### Peptides corresponding to the DEAD box helicase domain of CAGE decrease the migration, invasion and angiogenic potential of anti-cancer drug-resistant cancer cells

**Figure 8:**
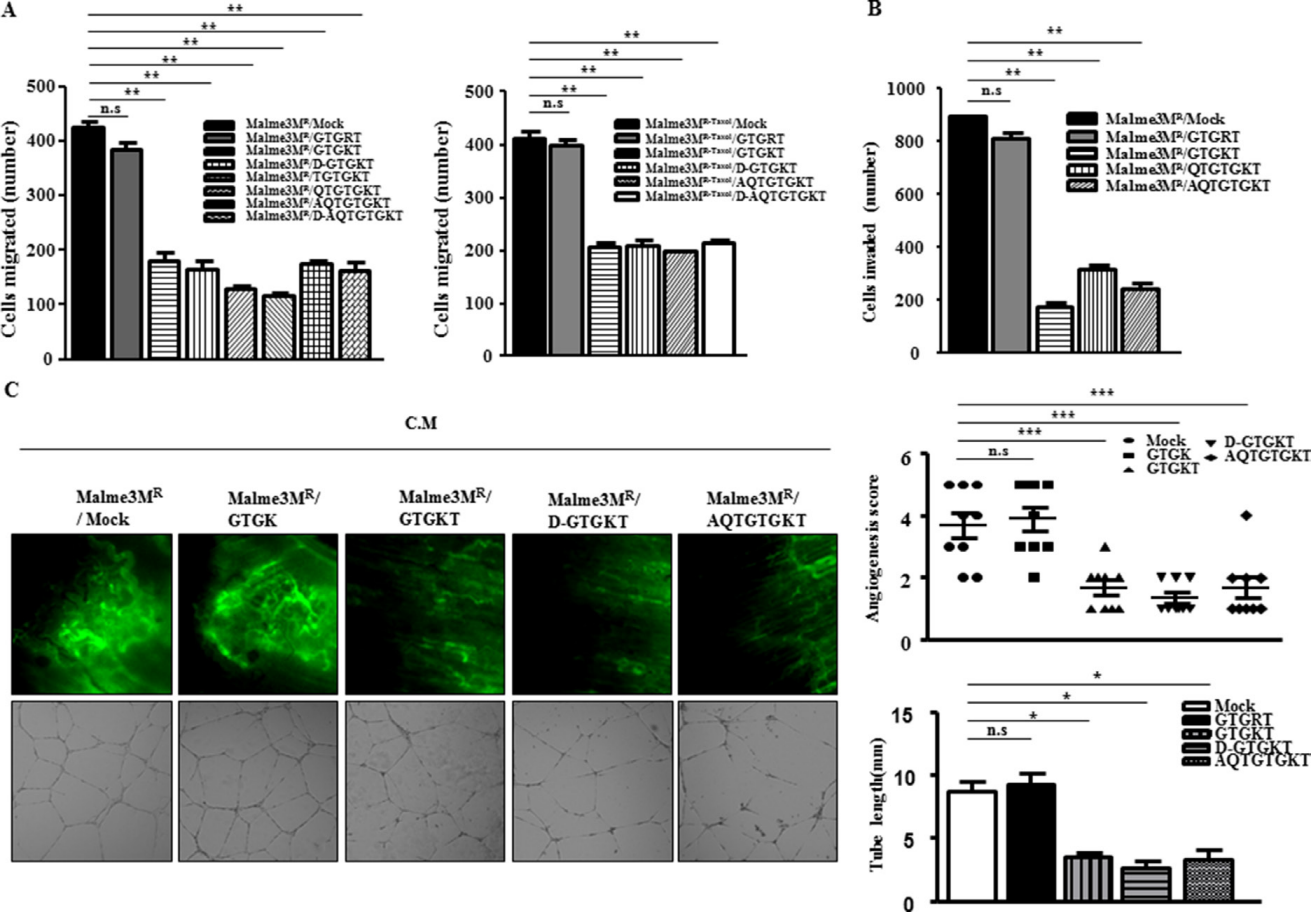
Peptides corresponding to the DEAD box helicase domain of CAGE decrease the migration, invasion and angiogenic potential of anti-cancer drug-resistant cancer cells (**A**) A scratch was made on the cell layer with a micropipette tip and cultures were washed twice with serum-free medium. Cells were then treated with peptide of interest (each at 10 μM). Wound migration assays were performed as described. **p < 0.005. N.S. denotes not significant. (**B**) Malme3M^R^ cells were treated with the indicated peptide (each at 10 μM). At 48 h after treatment, transwell invasion assays were performed as described. **p < 0.005. (**C**) Conditioned medium (C.M.) obtained from the Malme3M^R^ cells treated with the indicated peptide (each at 10 μM) was added to HUVECs. Eight hours after the addition of conditioned medium, intravital microscopy was performed as described (upper panel). The conditioned medium was also subjected to endothelial cell tube formation assays (lower panel). *p < 0.05; ***p < 0.0005.

Various peptides corresponding to the DEAD box helicase domain of CAGE, but not GTGRT peptide, decreased the migration potential (Figure 8A) and the invasion potential of Malme3M^R^ cells (Figure 8B). GSK3β decreases the angiogenesis by decreasing the expression of HIF-1α via phosphorylation and recruitment of ubiquitin ligase [25]. Recombinant CAGE protein displays angiogenic potential [8]. We examined the effect of peptides corresponding to the DEAD box helicase domain of CAGE on the angiogenic potential of anti-cancer drug-resistant cancer cells. The conditioned medium obtained from Malme3M^R^ cells treated with GTGKT, D-GTGKT or AQTGTGKT, but not treated with GTGK, inhibited the angiogenic potential of Malme3M^R^ cells based on intravital microscopy (Figure 8C). The conditioned medium of Malme3M^R^ cells treated with GTGKT, D-GTGKT or AQTGTGKT, when added to HUVECs, inhibited endothelial cell tube formation (Figure 8C). These results suggest that peptides corresponding to the DEAD box helicase domain of CAGE decrease the migration, invasion and angiogenic potential of cancer cells by binding to CAGE.

### AQTGTGKT peptide decreases the tumorigenic and metastatic potential of anti-cancer drug-resistant cancer cells

**Figure 9:**
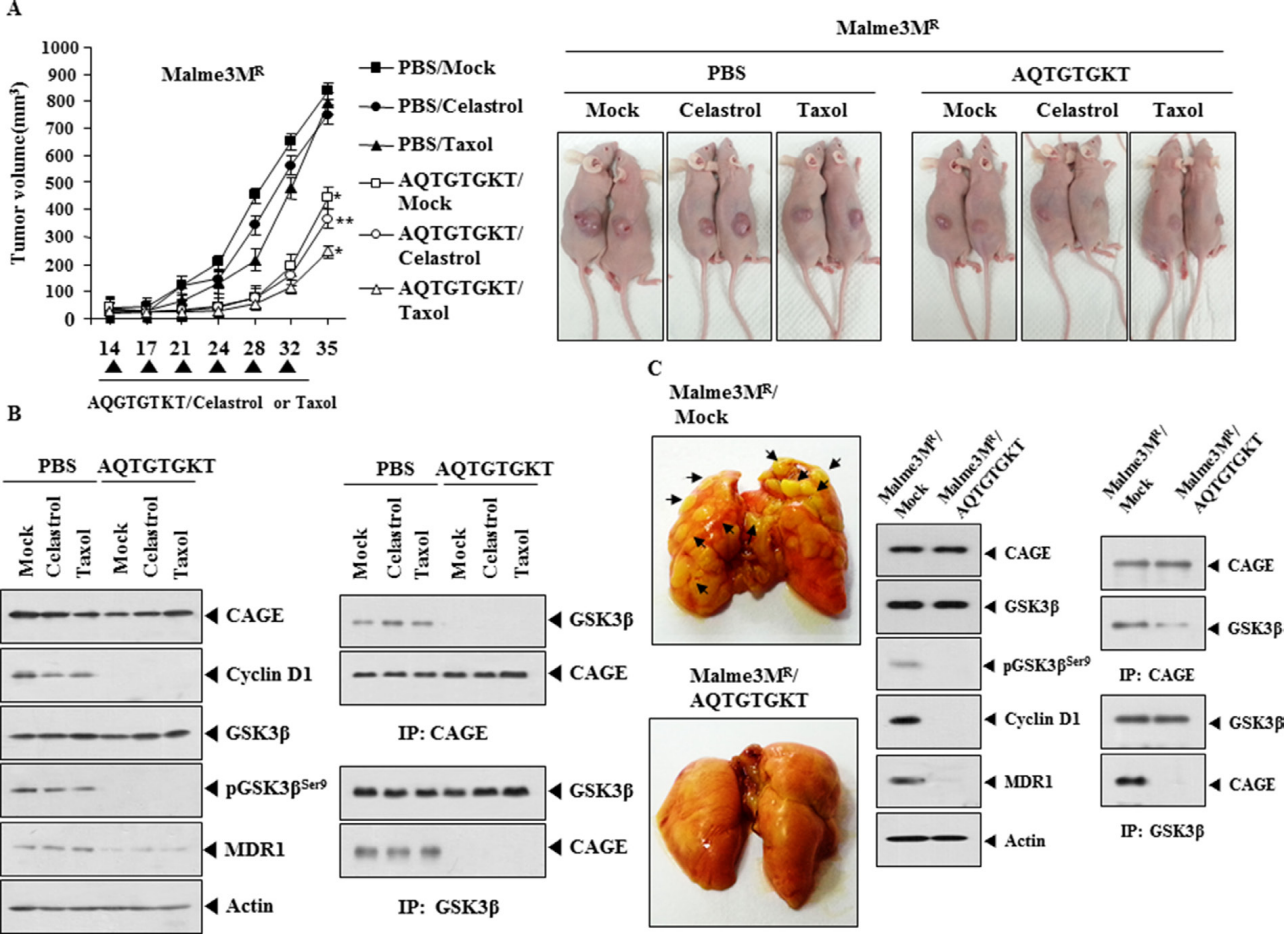
AQTGTGKT peptide decreases the tumorigenic and metastatic potential of anti-cancer drug-resistant cancer cells (**A**) Malme3M^R^ (1 × 10^6^) cells were injected into the dorsal flanks of athymic nude mice. Following the establishment of sizeable tumor, peptide (50 μg/mouse) was injected into the tail vein along with celastrol (1 mg /kg) or taxol (1 mg/kg) six times in a total of 35 days. Each experimental group consisted of five mice. Each value represents an average obtained from the five athymic nude mice of each group. Data are expressed as a mean ± S .D. Each figure shows a representative image of the mice in each group at the time of sacrifice. Statistically significant differences with PBS group are marked as *p < 0.05 and **p < 0.005, respectively. (**B**) Lysates from the indicated tumor tissues were subjected to Western blot analysis (left panel). Lysates were also immunoprecipitated with the indicated antibody (2 μg/ml), followed by Western blot analysis (right panel). (**C**) Each experimental group consists of five athymic nude mice. Each figure shows a representative image of the mice in each experimental group. AQTGTGKT peptide (50 μg/mouse) was intravenously injected five times over a total of 4 weeks. Tumor lysate from each mouse of the experimental group was immunoprecipitated with the indicated antibody (2 μg/ml), followed by Western blot analysis.

Because AQTGTGKT peptide exerted an anti-cancer activity *in vitro*, we examined the *in vivo* anti-cancer activity of AQTGTGKT peptide. AQTGTGKT peptide decreased the tumorigenic potential of Malme3M^R^ cells (Figure 9A). AQTGTGKT peptide enhanced the *in vivo* sensitivity of Malme3M^R^ cells to anti-cancer drugs (Figure 9A). AQTGTGKT peptide decreased the expression of cyclinD1, pGSK3β^Ser9^ and MDR1 (Figure 9B). AQTGTGKT peptide inhibited the binding of CAGE to GSK3β (Figure 9B). AQTGTGKT peptide decreased the metastatic potential of Malme3M^R^ cells in a manner associated with the inhibition of the binding of CAGE to GSK3β (Figure 9C). Western blot analysis of tumor lysates showed that AQTGTGKT peptide decreased the expression of cyclinD1, pGSK3β^Ser9^ and MDR1 (Figure 9C). Therefore the *in vivo* anti-cancer activity of AQTGTGKT peptide results from its effect on the expression of and cyclinD1 and the binding of CAGE to GSK3β.

### Peptides corresponding to the DEAD box helicase domain of CAGE enhance sensitivity to anti-cancer drugs in Malme3M cells transfected with miR-200b inhibitor

**Figure 10:**
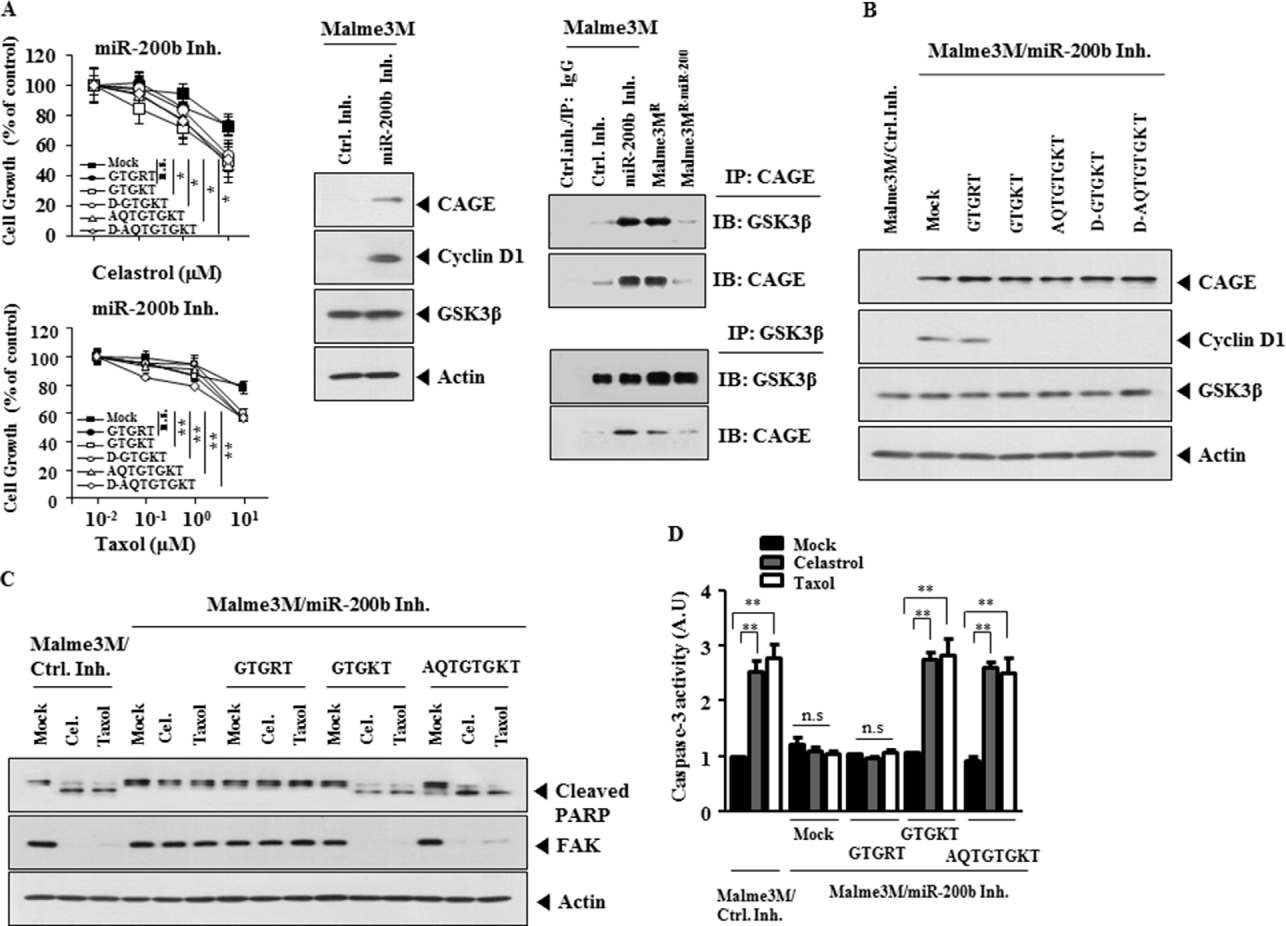
Peptides corresponding to the DEAD box helicase domain of CAGE enhance sensitivity to anti-cancer drugs in Malme3M cells transfected with miR-200b inhibitor (**A**) Malme3M cells were transiently transfected with control inhibitor or miR-200b inhibitor (each at 50 nM). At 24 h after transfection, cells were treated with various concentrations of celastrol or taxol for 24 h along with the indicated peptide (10 μM), followed by MTT assays (left panel). Statistically significant differences with mock control are marked as *p < 0.05, **p < 0.005 and n.s., not significant, respectively. Malme3M cells were transiently transfected with the indicated inhibitor (50 nM). At 48 h after transfection, cell lysates were subjected to Western blot analysis (middle panel). Cell lysates from Malme3M cells transfected with the indicated inhibitor (each at 50 nM) were immunoprecipitated with the indicated antibody (2 μg/ml), followed by Western blot analysis (right panel). Cell lysates from Malme3M^R^ or Malme3M^R-miR-200b^ were also immunoprecipitated with the indicated antibody (2 μg/ml), followed by Western blot analysis (right panel). (**B**) Malme3M cells were transiently transfected with the indicated inhibitor (each at 50 nM). At 24 h after transfection, cells were treated with the indicated peptide (10 μM) for 24 h, followed by Western blot analysis. (**C**) Malme3M cells were transiently transfected with control inhibitor or miR-200b inhibitor (each at 50 nM). At 24 h after transfection, cells were then transfected with celastrol (1 μM) or taxol (1 μM) along with each peptide (each at 10 μM) for 24 h, followed by Western blot analysis. (**D**) Same as C except that caspase-3 activity assays were performed. **p < 0.005.

miR-200b confers sensitivity to anti-cancer drugs by decreasing the expression of CAGE [8]. Peptides corresponding to the DEAD box helicase domain of CAGE, but not GTGRT peptide, prevented miR-200b inhibitor from conferring resistance to celastrol and taxol (Figure 10A). miR-200b inhibitor increased the expression of CAGE, cyclinD1 and induced the binding of CAGE to GSK3β (Figure 10A). Malme3M^R-miR-200b^ cells that stably express miR-200b did not show the binding of CAGE to GSK3β (Figure 10A). GTGKT, D-GTGKT, AQTGTGKT and D-AQTGTGKT peptides, but not GTGRT peptide, prevented miR-200b inhibitor from increasing the expression of cyclinD1 in Malme3M cells (Figure 10B). miR-200b inhibitor prevented cleavage of PARP and FAK in response to celastrol and taxol in Malme3M cells (Figure 10C). GTGKT and AQTGTGKT peptides, but not GTGRT peptide, enhanced the caspse-3 activity, in response to celastrol and taxol, in Malme3M cells transfected with miR-200b inhibitor (Figure 10D). These results confirm that GTGKT and AQTGTGKT peptides confer sensitivity to anti-cancer drugs by inactivation of CAGE.

### GTGKT peptide shows *ex vivo* tumor homing potential

**Figure 11:**
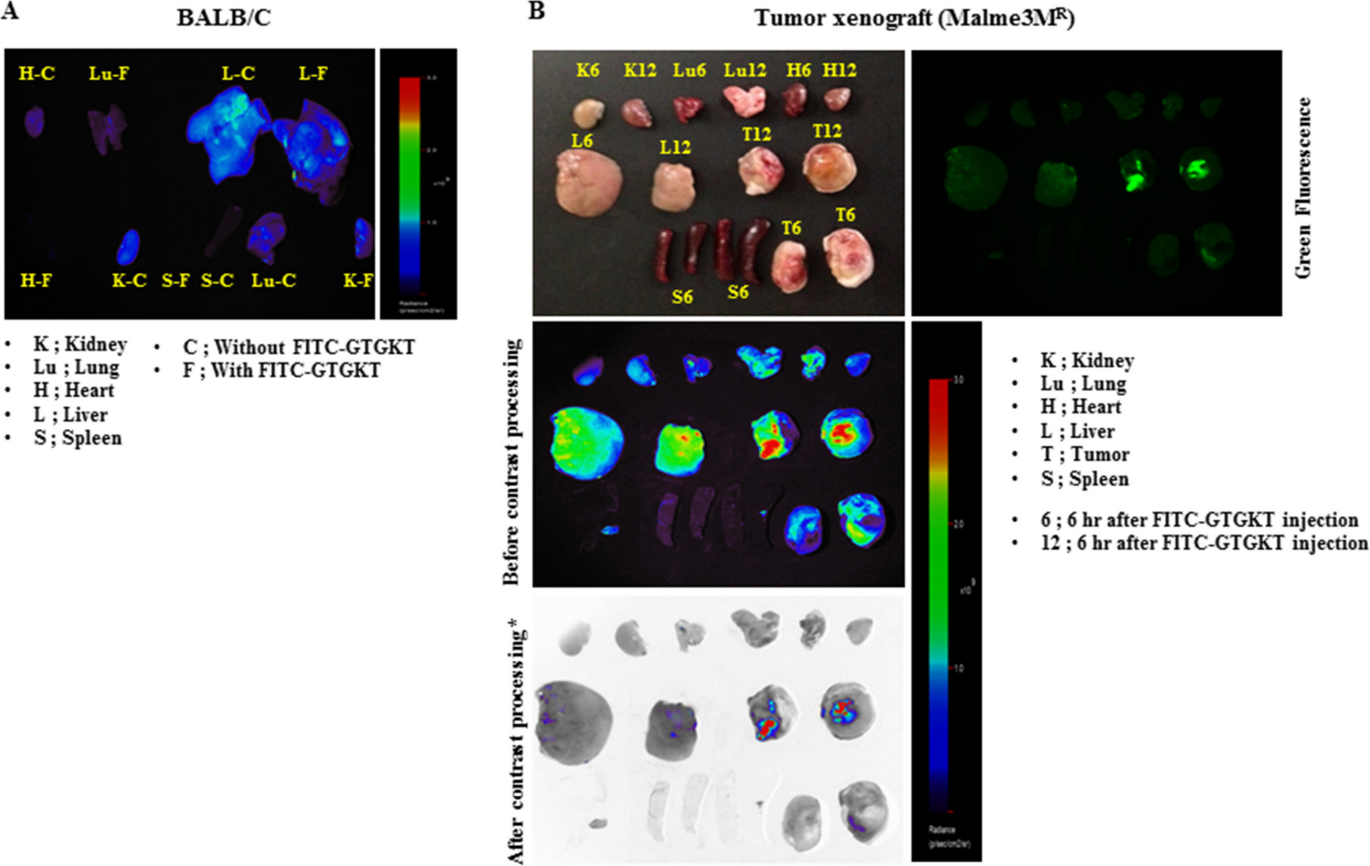
GTGKT peptide shows *ex vivo* tumor homing potential Tumor-bearing nude mice (xenograft of Malme3M^R^ cells) were given intravenous injection of 50 μg FITC-conjugated GTGKT peptide 4 weeks after Malme3M^R^ transplantation. Experimental and control mice were anesthetized and sacrificed 6 and 12 hrs later and the abdominal wall was opened and tumor and normal organs analyzed by confocal microscopy. The representative distribution of FITC-conjugated GTGKT peptide at 12 hrs after injection in dissected tumor and normal organs including kidney, liver, spleen, and lung is shown. Note the strong fluorescence accumulation in the tumor when compared to the control organs.

Because GTGKT peptide decreased the tumorigenic potential (Figure 4A) and the metastatic potential (Figure 5A) of Malme3M^R^ cells, we examined whether GTGKT peptide would display tumor homing potential. BALB/C mice injected with unlabeled GTGKT peptide or FITC-GTGKT peptide showed little fluorescence in any organs examined (Figure 11A). To determine the distribution of the FITC-conjugated GTGKT peptide in tumor bearing animals and examine whether FITC-conjugated GTGKT peptide localizes to tumor *in vivo*, tumor xenografts (Malme3M^R^ cells) were injected intravenously with FITC-conjugated GTGKT peptide. The peptide was then allowed to circulate in the blood stream for 6 and 12 hours before tumors and control organs were excised. Strong and specific fluorescence was detected in tumor xenografts (Malme3M^R^ cells) after 12 hrs injection with FITC-conjugated GTGKT peptide, while little labeling was seen in control organs (Figure 11B). These results show *ex vivo* tumor homing potential of GTGKT peptide.

## DISCUSSION

CyclinD1 promotes tumor growth and confers resistance to cisplatin in pancreatic cancer cell lines [26]. The expression level of cyclinD1 is higher in anti-cancer drug-resistant Malme3M^R^ and SNU387^R^ cells than in Malme3M and SUN387 cells, respectively (Supplementary Figure 1A). The expression level of phospho-cyclinD1^Thr286^ is lower in Malme3M^R^ and SNU387^R^ cells than in Malme3M and SUN387 cells, respectively (Supplementary Figure 1A). Based on the fact that the expression level of CAGE is higher in Malme3M^R^ and SNU387^R^ cells than in Malme3M and SUN387 cells, these results suggest the role of cyclinD1 in anti-cancer drug-resistance conferred by CAGE. It would be necessary to identify molecules functioning downstream of cyclinD1 for better understanding of anti-cancer drug-resistance conferred by CAGE. It would be also interesting to further identify miRNAs that decrease the expression of cyclinD1 and CAGE.

GSK3β accumulates in the nucleus and induces phosphorylation, nuclear export and subsequent degradation of cyclinD1 in the cytoplasm [27]. The phosphorylation of cyclinD1 at threonine 286 by glycogen synthase kinase 3 (GSK3) is required for the ubiquitination and nuclear export of cyclinD1 and its subsequent degradation in the proteasome [27, 28]. We show the nuclear localization of phospho-cyclinD1^Thr286^ in Malme3M cells, but not in Malme3M^R^ cells (Supplementary Figure 1B). This suggests that the phosphorylation of cyclinD1 at Thr 286 in Malme3M cells may be responsible for the lack of expression of cyclinD1 in Malme3M cells. We reported that SIAH2, an ubiquitin ligase, binds to and decreases the expression of HDAC3 in Malme3M^R^ cells [29]. It would be interesting to examine whether SIAH2 decreases the expression of cyclinD1 in Malme3M cells.

miR-145, miR-133a and miR-133b decrease the expression of the transcription factor SP1, knockdown of which reduces the expression of cyclinD1 [30]. It is therefore reasonable that these miRNAs decrease the expression of CAGE. NGF induces the expression of cyclinD1 via SP1 and NF-kB [31]. CAGE shows co-localization with SP1 (Figure 3A). SP1 shows the binding to the promoter sequences of cyclin D1 (Figure 3D). GTGKT peptide does not change the expression of CAGE. However, GTGKT peptide inhibits nuclear localization of CAGE and prevents CAGE from binding to the promoter sequences of cyclinD1 (Figure 3E). The disruption of HDAC2 suppresses the expression of cyclinD1, CDK4 and CDK2 [32]. Promoter analysis of cyclinD1 shows the binding site for HDAC2 (Supplementary Figure 3A). GTGKT peptide prevents HDAC2 from binding to the promoter sequences of cyclinD1 (Figure 3E). It is therefore probable that CAGE, through binding to HDAC2, may regulate the expression of cyclinD1.

miR-200b suppresses cell migration by inhibiting PKCα-Wnt 5 positive feedback loop [33]. Thus, CAGE may regulate PKCα-Wnt 5 positive feedback loop. It will be interesting to examine the effect of CAGE-miR-200b feedback loop on PKCα. In this study, we found an activation of PKCα in SNU387^R^ cells and an activation of PKCδ in Malme3M^R^ cells (personal observations). It would be interesting to examine the effect of PKCα and PKCδ on GSK3β activity and the expression and phosphorylation of cyclinD1. It would be also interesting to examine the effect of CAGE-derived peptides on PKC activity.

EGFR (epidermal growth factor receptor) activation leads to the up-regulation of PI3K/Akt signaling and cyclinD1 [34]. EGFR activation increases the phosphorylation of β-catenin and the expression of pGSK3β^Ser9^, resulting in a loss of β-catenin [35]. Malme3M^R^ cells show higher expression of pEGFR^Y845^ than Malme3M cells (personal observations). It is probable that CAGE activates EGFR signaling, which in turn increases the expression of pGSK3β^Ser9^ and cyclinD1. CAGE confers resistance to various EGFR inhibitors (personal observations). This implies that EGFR signaling is involved in anti-cancer drug-resistance conferred by CAGE. It would be necessary to examine the effect of peptides corresponding to the DEAD box helicase domain of CAGE on EGFR signaling in Malme3M^R^ cells. It will also be interesting to examine the possibility of the binding of CAGE to EGFR.

SBPs (self-binding peptides) are short peptides within monomeric proteins and bind to target domains to perform biological functions. SBPs are generally found in disordered or unstructured regions of monomeric proteins. Many of those protein-protein interactions are regulated by short peptides such as SBPs [36, 37]. Peptide drugs disrupt protein-protein interfaces to regulate biological processes [38]. ^100^RRNQYWV^106^ of c-Src serves as SBP and binds to PDZ5 domain of c-Src [39]. Given the fact that GTGKT peptide binds to CAGE, it is probable that GTGKT peptide can be considered as self-binding peptide (SBP). Given the fact that CAGE binds to GSK3β (Figure 1B), it is therefore reasonable that GTGKT peptide inhibits the binding of CAGE to GSK3β. GTGKT peptide binds to c-terminal domain of CAGE (Supplementary Figure 5A). It would be necessary to identify critical amino acids of CAGE that are necessary for binding to GTGKT peptide.

Anti-tumor peptides inhibit oncogenesis by binding to and inhibiting oncogenes with aberrant expression in cancer cells. For example, WT1 (Wilms tumor protein 1)-derived peptide binds to p53 and inhibits the binding of WT1 to p53 and enhances cellular senescence and decreases the metastatic potential of human melanoma cells [40]. Anti-tumor peptides induce apoptosis, block signaling mediators and receptors, and inhibit angiogenesis and the metastasis [41, 42]. GTGKT peptide displays tumor homing potential (Figure 11). PHSCNK peptide, a novel tumor homing peptide, acts as an antagonist of integrin α_5_β_1_ and suppresses melanoma [43]. CREKA peptide binds to fibrin and displays tumor homing potential [44]. It would be necessary to further identify receptor(s) of GTGKT peptide for better understanding of the mechanism of anti-cancer drug-resistance conferred by CAGE.

In this study, we show that peptides corresponding to the DEAD box helicase domain of CAGE can be developed as anti-cancer drugs for the treatment of cancer patients expressing CAGE.

## MATERIALS AND METHODS

### Chemicals and reagents

Chemicals used in this study were purchased from Sigma Chemical Company. Anti- mouse and anti-rabbit IgG-horse radish peroxidase conjugate antibody were purchased from Pierce Company (Rockford, IL). All other antibodies used in this study were purchased from Santa Cruz Company. Lipofectamine and PlusTM reagent for transfection were purchased from Invitrogen (San Diego, CA). Peptides used in this study were commercially synthesized by Peptron Company (Daejon, Koea). SiRNA kit was purchased from Ambion Company. Oligonucleotides used in this study were commercially synthesized by Bioneer Company (Daejon, Korea).

### Cell lines and cell culture

Cancer cell lines used in this study were cultured in Dulbecco's modified minimal essential medium (Invitrogen) supplemented with heat-inactivated 10% fetal bovine serum (Invitrogen) and antibiotics at 37°C in a humidified incubator with a mixture of 95% air and 5% CO2. Anti-cancer drug-resistant cancer cell lines (SNU387^R^ and Malme3M^R^) were established as described [6]. Malme3M^R^ and SNU387^R^ cell lines were established by stepwise addition of celastrol. Cells surviving drug treatment (attached fraction) were obtained and used throughout this study. Malme3M^R-taxol^ and SNU387^R-Taxol^ cell lines were established by stepwise addition of taxol. Human umbilical vein endothelial cells (HUVECs) were isolated from human umbilical cord veins by collagenase treatment and used in passages 3–6. The cells were grown in M199 medium supplemented with 20% fetal bovine serum, 100 units/ml penicillin G, 100 μg/ml streptomycin, 3 ng/ml bFGF (Upstate Biotechnology, Waltham, MA), and 5 units/ml heparin at 37 °C under 5% CO2, 95% air.

### Mice

All animal experiments were approved by Institutional Animal Care and Use Committee (IACUC) of Kangwon National University (KIACUC-14-0007).

### Peptides

CAGE-derived peptides were synthesized by Peptron Company, with the sequences GTGK, AQTGTGKT, QTGTGKT, TGTGKT, GTGKT, GTGRT, GTGKA, GTGAT, D-GTGKT, D-AQTGTGKT, SQWAP, RNGPG and a purity level > 95%. The FITC-conjugated GTGKT, FITC-conjugated AQTGTGKT, and Biotin-GTGKT peptide were purified by high performance liquid chromatography and their sequence and structure were confirmed by mass spectrometry.

### Preparation of siRNA duplexes and transfection

The siRNA duplexes were constructed with the following target sequences: CAGE, sense (5′-AACTCTGTCAACCTAAGAAGCCCTGTCTC-3′), and antisense (5′-AAGCTTCTTAGGTTGACAGAGCCTGTCTC-3′); CyclinD1, sense (5′-AACAGGTTCCACTTGAGCTTGCCTGTCTC 3′), and antisense (5′-AACAAGCTCAAGTGGAACCTGCCTGTCTC -3′); Scrambled CAGE, sense (5′-AA TTAATGATCGCCCAGAACC CCTGTCTC- 3′), and anti-sense (5′- AA GGTTCTGGGCGATCATTAA CCTGTCTC-3′); SP1, sense (5′-AAGACTCAATTCTGCTGCAAGCCTGTCTC- 3′ ), and antisense (5′- AACTTGCAGCAGAATTGAGTCCCTGTCTC3′ ); scrambled SP1, sense ((5′-AAAGTAAGGCCTACGATTCCTCCTGTCTC- 3′ ), and anti-sense (5′-AAGCGTACTATCGGCAATTGACCTGTCTC- 3′); Control, sense (5′-AATTCTCCGAACGTGTCACGTCCTGTCTC-3′), and antisense (5′-AAACGTGACACGTTCGGAGAACCTGTCTC-3′). Control siRNA sequences were derived from green fluorescent protein sequences. The construction of siRNAs was performed according to the instruction manual provided by the manufacturer (Ambion, Austin, TX). Transfections were performed according to the manufacturer's instructions. Lipofectamine and Plus reagents (Invitrogen) were used. The construction of siRNA was carried out according to the instruction manual provided by the manufacturer (Ambion, Austin, TX). For miR-200b knockdown, cells were transfected with 50 nM of oligonucleotide (inhibitor) with Lipofectamine 2000 (Invitrogen), according to the manufacturer's protocol. The sequences used were 5′-UCAUCAUUACCCAGUAUUA-3′ (miR-200b inhibitor) and 5′-GCAUAUAUCUAUUCCACUA-3′ (control inhibitor).

### CAGE constructs

Various CAGE deletion constructs were made by PCR amplification and cloning into Flag-tagged pcDNA3.1 vector. The full-length CAGE point mutant constructs were made by site-directed mutagenesis kit (Stratagene).

### Immunoprecipitation

Cells (1 × 10^7^) were lysed in immunoprecipitation buffer (50 mmol/liter HEPES, pH 7.6, 150 mmol/liter NaCl, 5 mmol/liter EDTA, 0.1% Nonidet P-40). After centrifugation (10 min at 15,000 × g) to remove particulate material, the supernatant was incubated with each antibody (2 μg/ml) with constant agitation at 4°C. The immune complexes were precipitated with protein A/G-Sepharose (Sigma) and analyzed by Western blot. To examine the effect of GTGKT peptide on the binding of CAGE to GSK3β, biotin-GTGKT peptide (Peptron, Daejeon, Korea) was transfected with Lipofectamin and PlusTM reagent (Invitrogen, San Diego, CA). After incubation for 48 h, whole-cell extracts were incubated with anti-biotin antibody (2 μg/ml) for 12 h at 4°C and immune complexes were precipitated with streptavidin-linked agarose beads for 30 min at 4°C. After five washes with lysis buffer, the bound proteins were eluted by boiling in 2X Laemli SDS loading buffer and were then subjected to SDS-PAGE followed by Western blotting analysis with anti-CAGE antibody.

### Western blot analysis

Western blot analysis was performed according to the standard procedures [8]. For analysis of proteins from tumor tissues, frozen samples were grounded to a fine powder using a mortar and pestle over liquid nitrogen. Proteins were solubilized in RIPA buffer containing protease inhibitors and insoluble material removed by centrifugation.

### Cell viability determination

The cells were assayed for their growth activity using the 3-(4, 5-dimethylthiazol-2-yl)-2, 5-diphenyltetrazolium bromide (MTT; Sigma). Viable cell number counting was carried out by trypan blue exclusion assays. At least two separate experiments were performed in triplicate.

### Colony formation

Cells were cultured at low density under treatment, and then colonies were stained with 0.01% crystal violet and counted.

### Caspase-3 activity assays

Caspase-3 activity was measured according to the manufacturer's instructions (BioVision, Palo Alto, CA). Cells were lysed in 0.1 M HEPES buffer, pH 7.4, containing 2 mM dithiothreitol, 0.1% CHAPS, and 1% sucrose. Cell lysates were incubated with a colorimetric substrate, 200 μM Ac-DEVD-*p*-nitroanilide, for 30 min at 30°C. The fluorescence was measured at 405 nm using a microtiter plate reader.

### Wound migration

Cells were plated overnight to achieve a confluent layer in 24-well plates. A scratch was made on the cell layer with a micropipette tip and cultures were washed twice with serum-free medium. Cells were then transfected with peptide of interest. Wound healing was visualized by comparing photographs taken at the time of transfection and 48 h later.

### Chemo invasion assays

The invasive potential was determined by using a transwell chamber system with 8-μm pore polycarbonate filter inserts (CoSTAR, Acton, MA). The lower and upper sides of the filter were coated with gelatin and Matrigel, respectively. Trypsinized cells (5 × 10^3^) in the serum-free RPMI 1640 medium containing 0.1% bovine serum albumin were added to each upper chamber of the transwell. RPMI 1640 medium supplemented with 10% fetal bovine serum was placed in the lower chamber and cells were incubated at 37°C for 16 h. The cells were fixed with methanol and the invaded cells were stained and counted. Results were analyzed for statistical significance using the Student's *t* test. Differences were considered significant when *p* < 0.05.

### Immunofluorescence staining

Cells were seeded on 10-mm coverslips at a density of 2 × 10^5^ cells/35-mm plate. Twenty-four hours after plating, cells were washed and fixed with 4% paraformaldehyde for 15 min at room temperature, and rinsed with cold PBS (pH 7.4). After blocking with goat serum (10%) in 0.1 % BSA/ PBS, primary antibodies to CAGE (Santa Cruz, 1:100), GSK3β (Santa Cruz, 1:100), pGSK3β^Ser9^ (Santa Cruz, 1:100), SP1 (Santa Cruz, 1:100), and phospho-cyclinD1^Thr286^ (Cell signaling Technology, 1:200) were added and cells were incubated at 4°C for 24 hour. After washing with PBS, slides were incubated with anti-rabbit Alexa Fluor 488 (for pcyclin D^Thr286^), anti-mouse Alexa Fluor 488 (for CAGE), or anti-goat Alexa Fluor 568 (pGSK3β^Ser9^, GSK3β, SP1) secondary antibodies for 1.5 hours at RT. After removal of antibodies, cells were washed with PBS and stained with DAPI and mounted with mounting medium. Fluorescence staining was visualized using confocal microscopy.

### Internalization experiments

We used confocal microscopy to evaluate whether the FITC-conjugated-GTGKT and FITC-AQTGTGKT peptides were able to internalize. Briefly, Malme3M^R^ cells (3 × 10^5^) cells were seeded in 35mm culture dishes. After 24 hours of incubation, medium was replaced by fresh medium and FITC-conjugated GTGKT peptide (10 μM) or FITC-conjugated AQTGTGKT peptide (10 μM) was added to the cells. The FITC- GTGKT or FITC-AQTGTGKT peptide was incubated with the tumor cells for various time intervals. The medium was then removed and the cells were washed with 1 ml PBS before being analyzed by confocal imaging on an inverted microscope with a confocal laser scanning unit. Unlabeled GTGKT peptide or AQTGTGKT peptide was employed as negative control to determine auto fluorescence of tumor cells. Processed serial sections were constructed into three-dimensional images using VoxelView (Vital Images Ltd., Fairfield, IA) on a Silicon Graphics Indy workstation (Mountain View, CA).

### Chromatin immunoprecipitation assays (ChIP)

Assays were performed according to manufacturer's instruction (Upstate). For detection of the binding of protein of interest to cyclinD1 promoter sequences, specific primers of cyclinD1 promoter-1 sequences [5′-CCCAACTGCACCCCCTCCCT -3′ (sense) and 5′-AGCCCCCTCACGCTCACGAA -3′ (antisense)], cyclinD1 promoter-2 sequences [5′- GACCGGGCACACAACCCCTG -3′ (sense) and 5′- CAGTTGGGGACCCCCGCAAG -3′ (antisense)] and cyclinD1 promoter-3 sequences [5′- TTGCAGCTCGCCGTGCTCTC-3′ (sense) and 5′-GTGCCAGGGGCACCCCAATG -3′ (antisense)] were used.

### Intravital microscopy

Male BALB/c mice (6–8 week old) were obtained from Daehan Biolink (Korea). *In vivo* angiogenesis was assessed as follows. The mice were anesthetized with 2.5% avertin (v/v) via intraperitoneal injection (Surgivet, USA), and abdominal wall windows were implanted. Next, a titanium circular mount with eight holes on the edge was inserted between the skin and the abdominal wall. Growth factor-reduced matrigel containing the conditioned medium was applied to the space between the windows, and a circular glass cover slip was placed on top and fixed with a snap ring. After four days, the animals were anesthetized and injected intravenously with 50 μl of 25 ng/ml fluorescein isothiocyanate-labeled dextran (molecular weight, Mr ~2,000,000) via the tail vein. The mice were then placed on a Zeiss Axiovert 200 M microscope. The epi-illumination microscopy setup included a 100-W mercury lamp and filter set for blue light. Fluorescence images were recorded at random locations of each window using an electron-multiplying charge coupled device camera (Photo Max 512, Princeton Instruments, USA) and digitalized for subsequent analysis using the Metamorph program (Universal Imaging, USA). The assay was scored from 0 (negative) to 5 (most positive) in a double-blinded manner.

### Endothelial cell tube formation assays

Growth factor–reduced matrigel was pipetted into pre-chilled 24-well plates (200 μl matrigel per well) and polymerized for 30 min at 37°C. The HUVECs were placed onto the layer of matrigel in 1 ml of M199 containing 1% FBS. After 6 to 8 h of incubation at 37°C in a 95%:5% (v/v) mixture of air and CO_2_, the endothelial cells were photographed using an inverted microscope (magnification, X100; Olympus). Tube formation was observed using an inverted phase contrast microscope. Images were captured with a video graphic system. The degree of tube formation was quantified by measuring the length of tubes in five randomly chosen low-power fields (×100) from each well using the Image-Pro plus v4.5 (Media Cybernetics, San Diego, CA, USA).

### *In vivo* tumorigenic potential

Athymic nude mice (BALB/c nu/nu, 5–6-week-old females) were obtained from Orient Bio Inc. (Seoul, Korea) and were maintained in a laminar air-flow cabinet under aseptic conditions. Cancer cells (1 × 10^6^) of interest were injected subcutaneously into the dorsal flank area of the mice. Tumor volume was determined by direct measurement with calipers and calculated by the following formula: length × width × height × 0.5. To determine the effect of peptides corresponding to the DEAD box helicase domain of CAGE on the tumorigenic potential, each peptide (10 μg/mouse, 50 μg/mouse, or 0.6 mg/kg, 3 mg/kg) was injected via tail vein five times in a total of 35 days or four times in a total of 35 days. To determine the effect of CAGE on the tumorigenic potential and *in vivo* anti-cancer drug-resistance, taxol (1 mg/kg) was injected, following the establishment of sizable tumor by injection of Malme3M^R-As-CAGE^ cells (1 × 10^6^) or Malme3M^R-Vector^ cells, via tail vein four times in a total of 30 days.

### *In vivo* metastasis assay

Female athymic nude mice were used for the studies. Malme3M^R^ cells (1 × 10^6^ cells in PBS) were injected intravenously into the tail vein of 4-week-old athymic nude mice, and the extent of lung metastasis was evaluated. Peptide (50 μg/mouse) of interests was injected intravenously into the tail vein of athymic nude mice five times. Four weeks after injection of cancer cells, surface metastatic nodules per lung were determined.

### *Ex vivo* homing assays

Athymic nude mice (BALB/c nu/nu, 5–6-week-old females) were obtained from Orient Bio Inc. (Seoul, Korea) and were maintained in a laminar air-flow cabinet under aseptic conditions. Malme3M^R^ cells (1 × 10^6^) were injected subcutaneously into the dorsal flank area of the mice. Four-weeks after the injection of Malme3M^R^ cells, tumor bearing mice were given intravenous injection of 50 μg FITC-GTGKT peptide. FITC-GTGKT peptide was allowed to circulate for 6 and 12 hours. In some experiments mice were perfused at the time of sacrifice with PBS through the left ventricle to remove blood and unbound peptide. Tumors and control organs were excised after the injection of the fluorescent peptide and examined for fluorescence using a versatile bio-imaging system (Davinch-*In vivo* Imaging System; Davinch-K, Seoul, Korea). Images were acquired using Davinch *in vivo* imaging system with excitation at 490 nm and the emitted fluorescence was collected through a long-pass filter (520 nm). Data were analyzed by Davinch *In vivo* software (Davinch-K, Seoul, Korea).

### Statistical analysis

Statistical differences in this were determined by using the Student's *t* test. *P* ≤ 0.05 was considered statistically significant.

## SUPPLEMENTARY MATERIALS FIGURES


