# Supplementary material for: The pentapeptide Gly-Thr-Gly-Lys-Thr confers sensitivity to anti-cancer drugs by inhibition of CAGE binding to GSK3β and decreasing the expression of cyclinD1
Source: Oncotarget. 2017 Jan 13;8(8):13632–51. doi: 10.18632/oncotarget.14621 (PMC5355126; doi:10.18632/oncotarget.14621)
Supplement: Supplementary file 1 [file oncotarget-08-13632-s001.pdf]

The pentapeptide Gly-Thr-Gly-Lys-Thr confers sensitivity to anti-cancer drugs by inhibition of CAGE binding to GSK3β and decreasing the expression of cyclinD1

Supplementary Materials

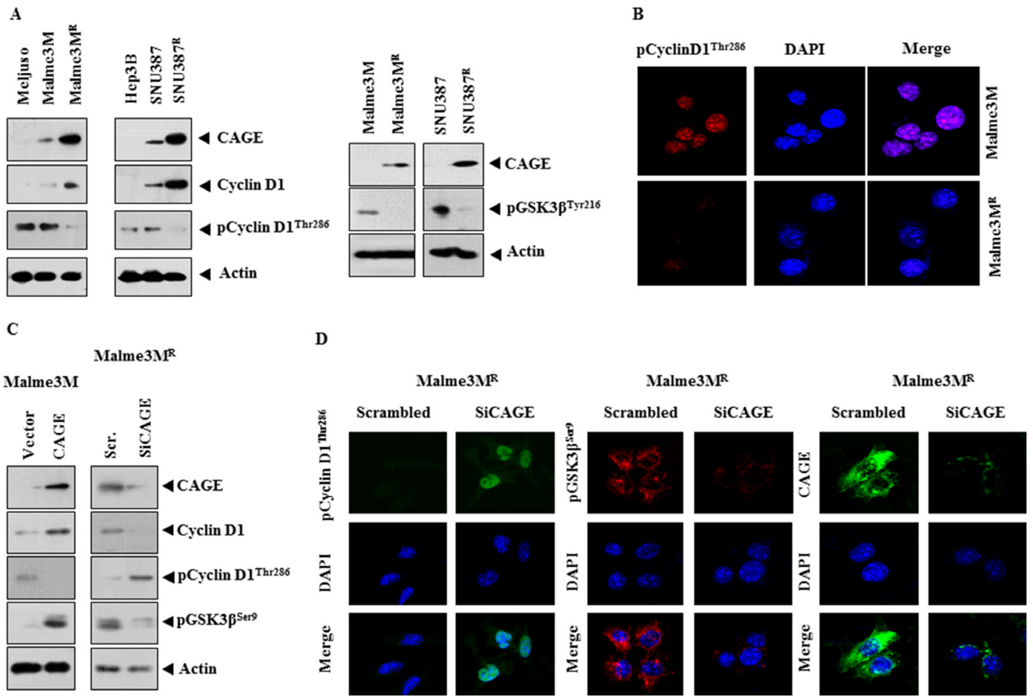

Supplementary Figure 1: CAGE increases the expression of cyclin D1 and pGSK3β^{Ser9} while decreasing the expression of phospho-cyclinD1^{Thr286}. (**A**) Cell lysates isolated from the indicated cancer cell lines were subjected to Western blot analysis. (**B**) Immunofluorescence staining employing the indicated antibody was performed. (**C**) Malme3M cells were transfected with control vector (1 µg) or CAGE (1 µg). At 48 h after transfection, cell lysates were subjected to Western blot analysis (left panel). Malme3M^R cells were transfected with the indicated siRNA (10 nM). At 48 h after transfection, cell lysates were subjected to Western blot analysis (right panel). (**D**) Malme3M^R cells were transfected with the indicated siRNA (10 nM). At 48 h after transfection, immunofluorescence staining employing the indicated antibody was performed.

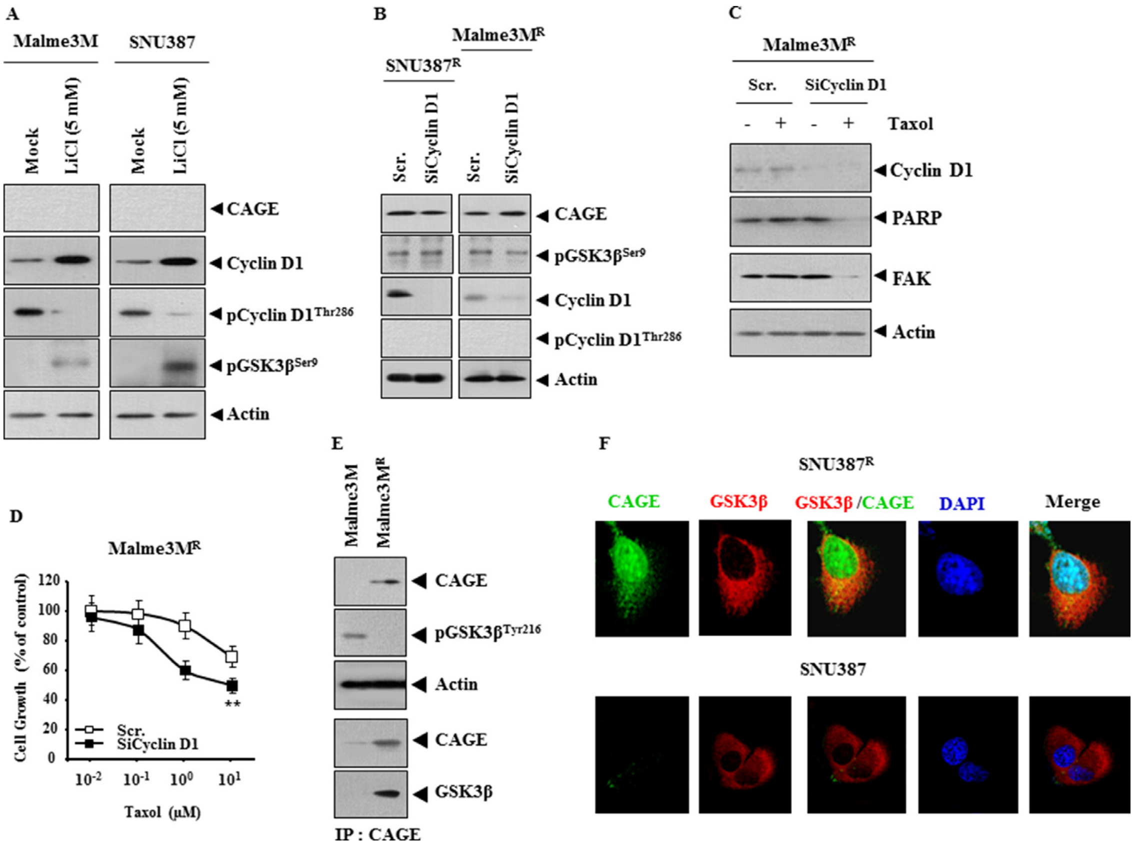

Supplementary Figure 2: GSK3β-CyclinD1 axis functions downstream of CAGE. (**A**) The indicated cancer cells were treated with or without LiCl (5 mM) for 12 h. Cell lysates were subjected to Western blot analysis. (**B**) Malme3M^{R} or SNU387^{R} cells were transiently transfected with the indicated siRNA (each at 10 nM). At 48 h after transfection, cell lysates were subjected to Western blot analysis. (**C**) Malme3M^{R} cells were transiently transfected with the indicated siRNA (each at 10 nM). The next day, cells were then treated with taxol (1 μM) for 24 h, followed by Western blot analysis. (**D**) The indicated cancer cells were transiently transfected with the indicated siRNA (each at 10 nM). The next day, cells were then treated with various concentrations of taxol for 24 h, followed by MTT assays. The mean ± S.E. of three independent experiments is shown. $^{**}p < 0.005$. (**E**) Cell lysates from the indicated cancer cells were immunoprecipitated with the indicated antibody (2 μg/ml), followed by Western blot analysis. (**F**) Immunofluorescence staining employing the indicated antibody was performed. DAPI staining was also performed to determine the integrity of the indicated cancer cells.

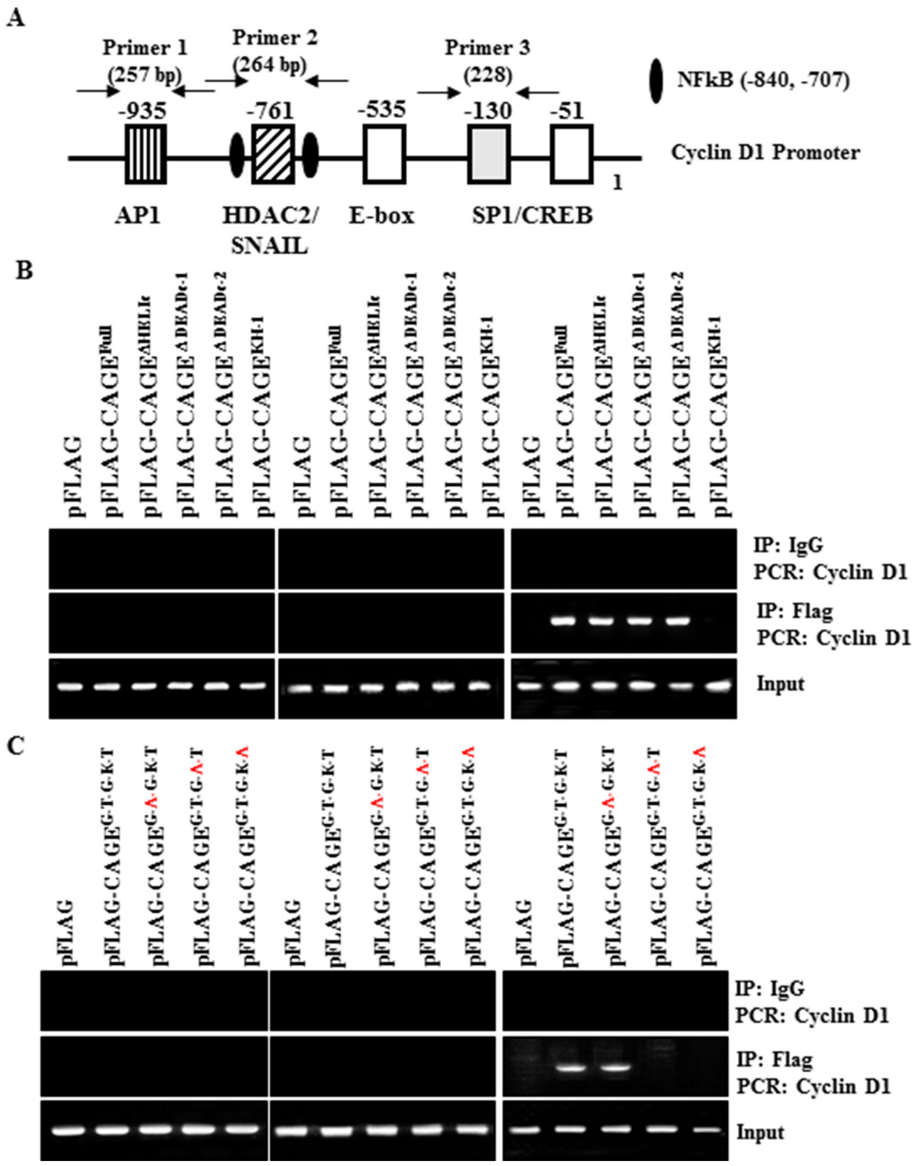

Supplementary Figure 3: KH-1 domain of CAGE is necessary for the binding to the promoter sequences of cyclinD1. (**A**) Shows potential binding sites of transcriptional factors in the promoter sequences of cyclinD1. (**B**) and (**C**) Malme3M cells were transiently transfected with the indicated construct (each at 1 μg). At 48 h after transfection, cell lysates were subjected to ChIP assays.

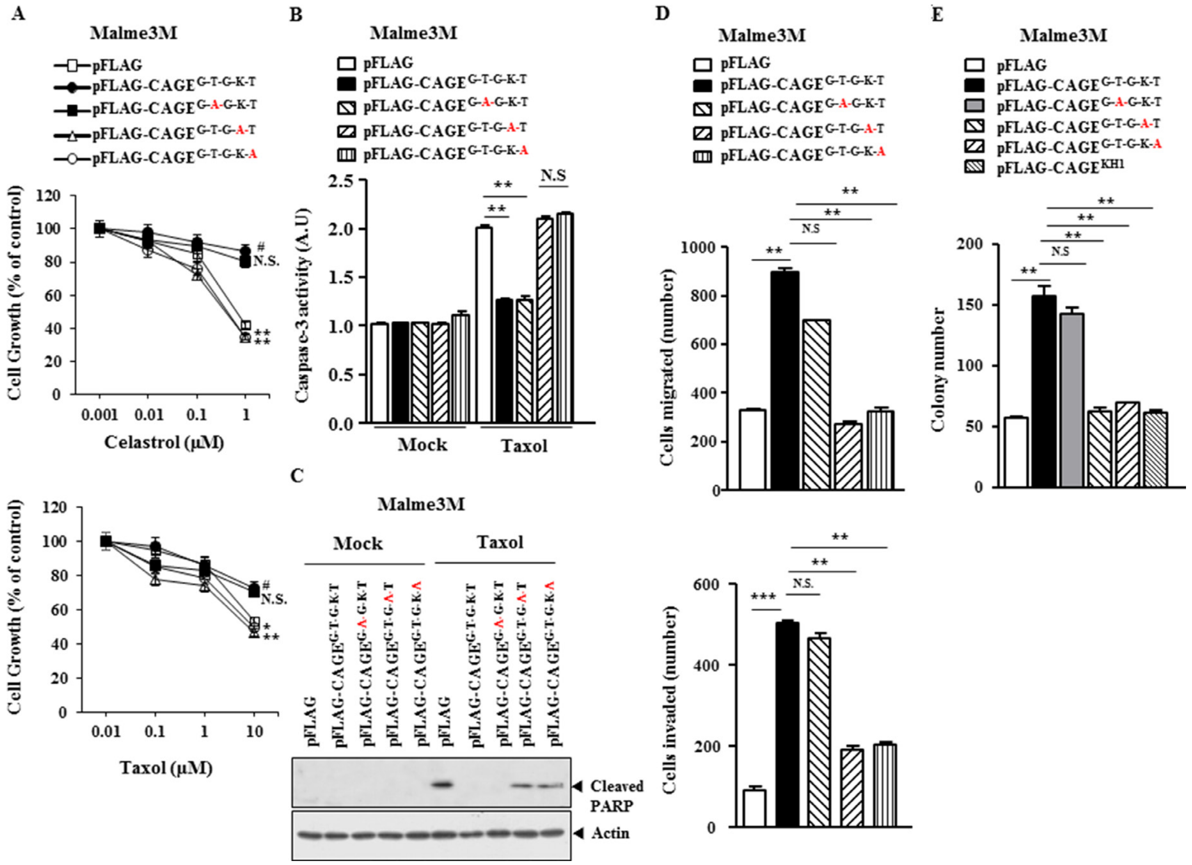

Supplementary Figure 4: Full-length CAGE mutants (G-T-G-A-T and G-T-G-K-A) do not confer resistance to anti-cancer drugs. (**A**) Malme3M cells were transfected with the indicated with the construct (each at 1 μg). The next day, cells were then treated with various concentrations of celastrol or taxol for 24 h, followed by MTT assays. $^{\#}p < 0.05$; $^{*}p < 0.05$; $^{**}p < 0.005$. #; comparison was made between Malme3M cells transfected with pFLAG-CAGE^{Full} and the same cells transfected with pFLAG. *, **; comparison was made between Malme3M cells transfected with pFLAG-CAGE$^{\text{G-T-G-A-T}}$ or pFLAG-CAGE$^{\text{G-T-G-K-A}}$ and the same cells transfected with pFLAG-CAGE^{Full}. N.S.; comparison was made between Malme3M cells transfected with pFLAG-CAGE$^{\text{G-A-G-K-T}}$ and the same cells transfected with pFLAG-CAGE^{Full}. (**B**) Malme3M cell were transfected with the indicated with the construct (each at 1 μg). The next day, cells were then treated with taxol (1 μM) for 24 h, followed by caspase-3 activity assays. $^{*}p < 0.005$. (**C**) Same as (B) except that Western blot analysis was performed. (**D**) Malme3M cell were transfected with the indicated with the construct (each at 1μg). At 48 h after transfection, cells were subjected to migration (upper panel) and invasion assays (lower panel). $^{**}p < 0.005$; $^{***}p < 0.0005$. (**E**) Malme3M cells were transiently transfected with the indicated construct (each at 1 μg). At 48 h after transfection, coly formation assay were performed using 100 cells. $^{**}p < 0.005$.

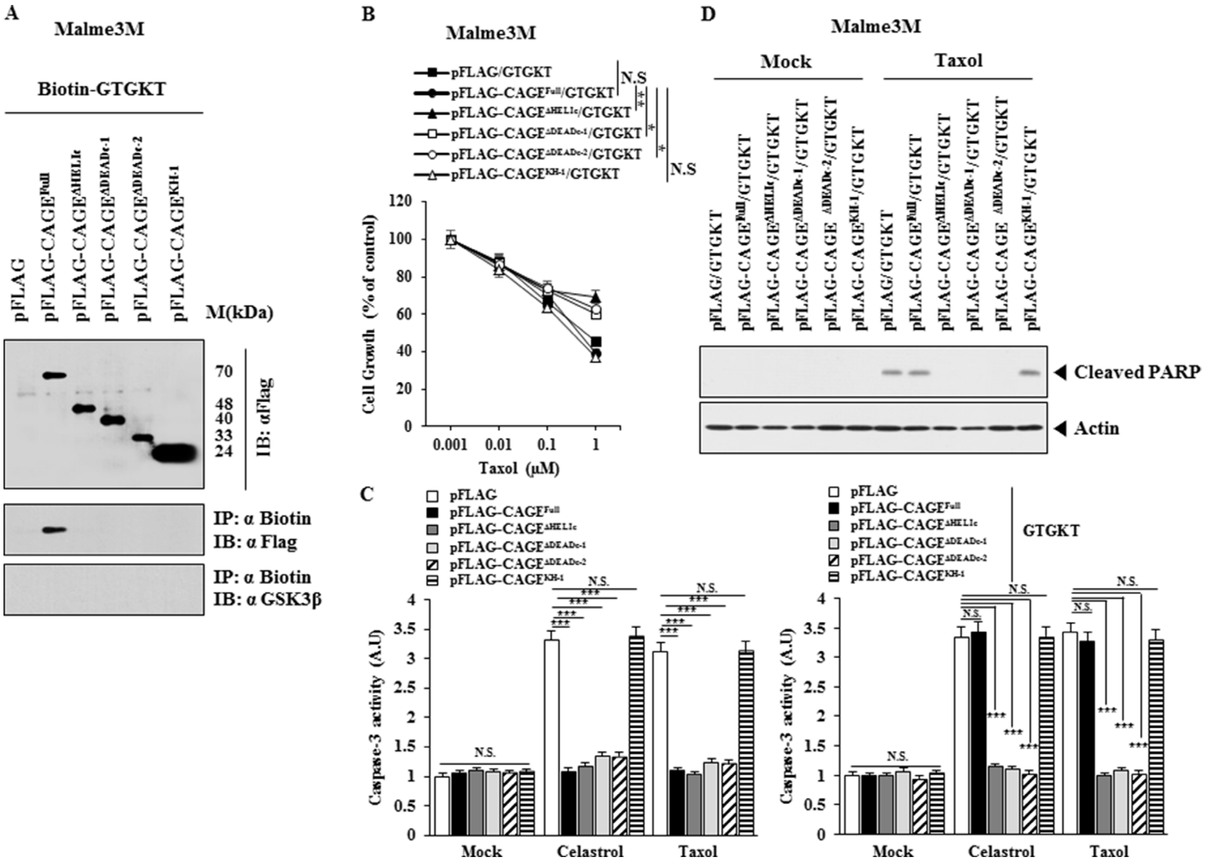

Supplementary Figure 5: The binding of GTGKT peptide to CAGE is necessary for conferring sensitivity to anti-cancer drugs. (**A**) Malme3M cells were transiently transfected with the indicated construct (each at 1 μg). The next day, cells were then treated with biotin-GTGKT peptide (10 μM). At 24 h after treatment with biotin-GTGKT peptide, Western blot and immunoprecipitation were performed. (**B**) Malme3M cells were transiently transfected with the indicated construct (each at 1 μg). The next day, cells were then treated with GTGKT peptide (10 μM) along with taxol at the indicated concentration. At 24 h after treatment, MTT assays were performed. *$p < 0.05$; **$p < 0.005$. (**C**) Malme3M cells were transiently transfected with the indicated construct (each at 1 μg). The next day, cells were then treated without or with GTGKT peptide (10 μM) along with celastrol (1 μM) or taxol (1 μM). At 24 h after treatment, caspase-3 activity assays were performed. ***$p < 0.0005$. (**D**) Malme3M cells were transiently transfected with the indicated construct (each at 1 μg). The next day, cells were then treated without or with GTGKT peptide (10 μM) along with taxol (1 μM). At 24 h after treatment, Western blot analysis was performed.

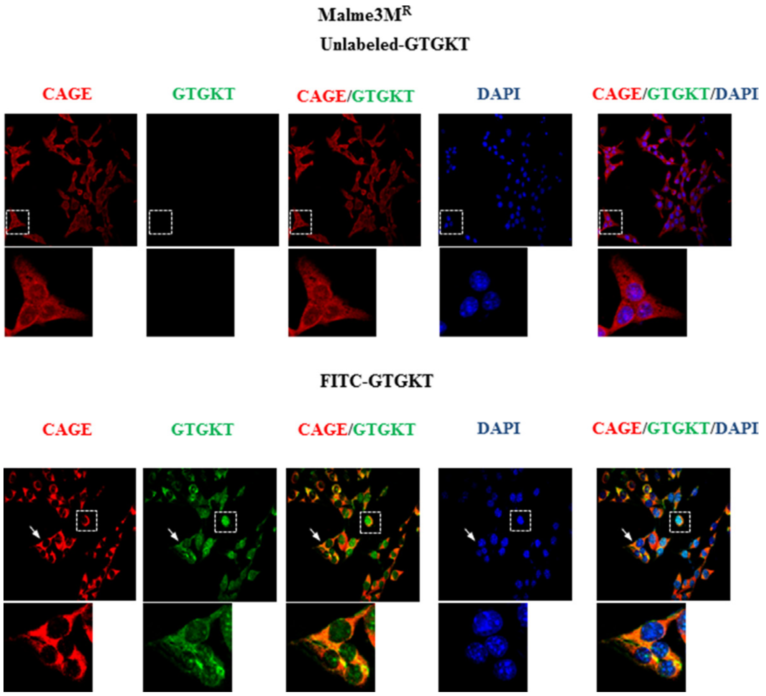

Supplementary Figure 6: GTGKT peptide shows co-localization with CAGE. FITC-labeled GTGKT peptide (5, 10 μM) or unlabeled GTGKT peptide (10 μM) was added into Malme3M^R cells. At 24 h after treatment, co-localization of GTGKT peptide with CAGE was examined.

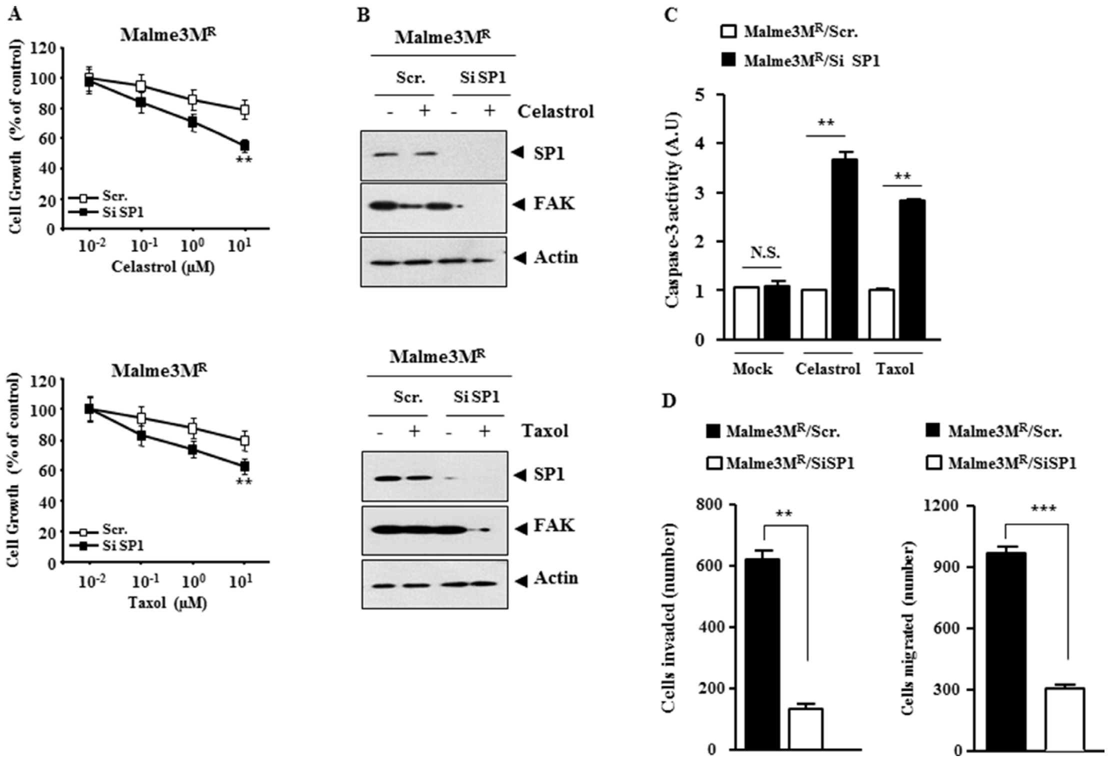

Supplementary Figure 7: The down-regulation of SP1 enhances sensitivity to anti-cancer drugs in Malme3M^R cells. (**A**) The indicated cancer cells were transiently transfected with the indicated siRNA (each at 10 nM). The next day, cells were then treated with various concentrations of celastrol or taxol for 24 h, followed by MTT assays. $^{**}p < 0.005$. (**B**) The indicated cancer cells were transiently transfected with the indicated siRNA (each at 10 nM). The next day, cells were then treated with celastrol (1 μM) or taxol (1 μM) for 24 h, followed by Western blot analysis. (**C**) Same as (B) except that caspase-3 activity assays were performed. $^{**}p < 0.005$. (**D**) The indicated cancer cells were transiently transfected with the indicated siRNA (each at 10 nM). At 48 h after transfection, cells were subjected to invasion and migration assays. $^{**}p < 0.005$; $^{***}p < 0.0005$.

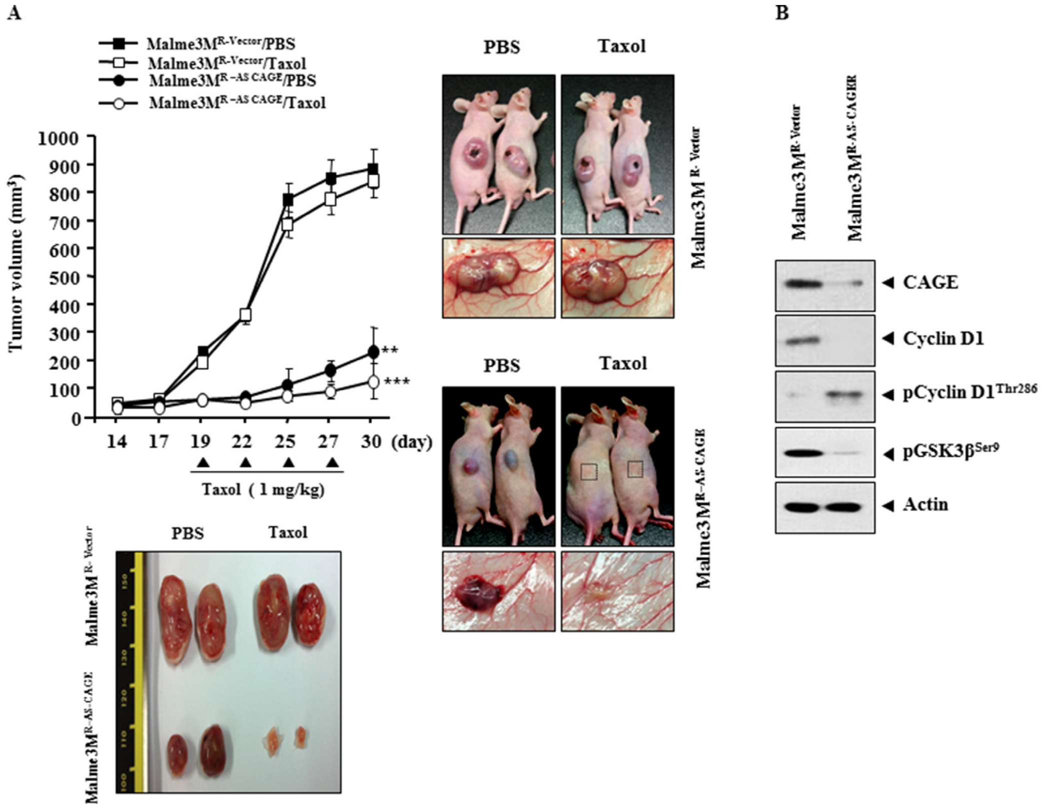

Supplementary Figure 8: The downregulation of CAGE confers sensitivity to anti-cancer drug *in vivo*. (**A**) Malme3M^{Vector} (1×10^6) or Malme3M$^{AS\text{-}CAGE}$ cells (1×10^6) were injected into the dorsal flanks of athymic nude mice. Taxol (1 mg/kg) was injected into each nude mouse after the tumor reached a certain size. Tumor volume was measured as described. Each experimental group consists of five mice. Each value represents an average obtained from five athymic nude mice of each group. Data are expressed as mean ± S.D. Statistically significant differences with PBS group are marked as $**p < 0.005$ and $***p < 0.0005$, respectively. (**B**) Tumor tissue lysates were subjected to Western blot analysis.
